# Exploring the mialome of ticks: an annotated catalogue of midgut transcripts from the hard tick, *Dermacentor variabilis *(Acari: Ixodidae)

**DOI:** 10.1186/1471-2164-9-552

**Published:** 2008-11-20

**Authors:** Jennifer M Anderson, Daniel E Sonenshine, Jesus G Valenzuela

**Affiliations:** 1 Vector Molecular Biology Unit, Laboratory of Malaria and Vector Research, National Institutes of Allergy and Infectious Diseases, National Institutes of Health, Rockville, Maryland 20852, USA; 2 Department of Biological Sciences, Old Dominion University, Norfolk, Virginia 23529, USA

## Abstract

**Background:**

Ticks are obligate blood feeders. The midgut is the first major region of the body where blood and microbes ingested with the blood meal come in contact with the tick's internal tissues. Little is known about protein expression in the digestive tract of ticks. In this study, for analysis of global gene expression during tick attachment and feeding, we generated and sequenced 1,679 random transcripts (ESTs) from cDNA libraries from the midguts of female ticks at varying stages of feeding.

**Results:**

Sequence analysis of the 1,679 ESTs resulted in the identification of 835 distinct transcripts, from these, a total of 82 transcripts were identified as proteins putatively directly involved in blood meal digestion, including enzymes involved in oxidative stress reduction/antimicrobial activity/detoxification, peptidase inhibitors, protein digestion (cysteine-, aspartic-, serine-, and metallo-peptidases), cell, protein and lipid binding including mucins and iron/heme metabolism and transport. A lectin-like protein with a high match to lectins in other tick species, allergen-like proteins and surface antigens important in pathogen recognition and/or antimicrobial activity were also found. Furthermore, midguts collected from the 6-day-fed ticks expressed twice as many transcripts involved in bloodmeal processing as midguts from unfed/2-day-fed ticks.

**Conclusion:**

This tissue-specific transcriptome analysis provides an opportunity to examine the global expression of transcripts in the tick midgut and to compare the gut response to host attachment versus blood feeding and digestion. In contrast to those in salivary glands of other Ixodid ticks, most proteins in the *D. variabilis *midgut cDNA library were intracellular. Of the total ESTs associated with a function, an unusually large number of transcripts were associated with peptidases, cell, lipid and protein binding, and oxidative stress or detoxification. Presumably, this is consistent with their role in intracellular processing of the blood meal and response to microbial infections. The presence of many proteins with similar functions is consistent with the hypothesis that gene duplication contributed to the successful adaptation of ticks to hematophagy. Furthermore, these transcripts may be useful to scientists investigating the role of the tick midgut in blood-meal digestion, antimicrobial activity or the transmission of tick-borne pathogens.

## Background

Ticks are notorious as vectors of a large variety of infectious disease agents such as those that cause Lyme disease, Rocky Mountain spotted fever, human (and animal) anaplasmosis, babesiosis and many others. As obligate hematophagic parasites, tick-borne infectious agents are acquired during the blood-feeding process. The tick midgut is the first tissue that pathogens encounter where infection must be established before they can migrate into the internal body organs and tissues. The effects of digestive enzymes expressed in response to the nutritive elements, especially hemoglobin, and the products of their digestive activities have been shown to inhibit or kill some invading microbes [1-3]. However, whether such peptidic fragments would also kill pathogenic microbes is unknown. In addition, antimicrobial peptides defensin and lysozyme have been reported from the midgut of a soft tick [4,5] and evidence of their expression (but not the peptides) was found in the hard tick *Dermacentor variabilis*. Although few in number, these reports suggest that the tick midgut presents a hostile environment for invading microorganisms. Nevertheless, many survive and succeed in colonising this tissue and penetrating the tick internal organs.

In blood-feeding insects, molecules expressed in the midgut of these disease-transmitting vectors are believed to play an important role in determining initial parasite infection and subsequent development. In tsetse flies, for example, 17 midgut proteins were upregulated and 9 were downregulated in response to blood-meal intake [6]. In mosquitoes, blood feeding induces expression of midgut peptidases such as trypsin, chymotrypsin, aminopeptidase, and carboxypeptidase; enzymes that may contribute to success in *Plasmodium *infection [7,8]. Changes in midgut transcript expression after a blood meal were also recently reported from the midgut of the sand fly *Phlebotomus papatasi *[9].

Knowledge of the tick digestive process is even more limited. The primary nutritive element, hemoglobin, is digested intracellularly in acidic phagolysosomes of the digestion cells. Most of the heme is shuttled to these specialised organelles, designated as hemosomes, where this highly reactive species is detoxified to hematin [10,11], a non-crystalline heme aggregate similar (but not identical) to malarial hemozoin [12]. Acid phosphatase and non-specific esterases have been reported to occur in the midgut of several hard-tick species [13,14] but their molecular structure was not identified. More recently, a cysteine peptidase was identified in the midguts of cattle ticks, *Rhipicephalus (Boophilus) microplus *[15], while serine peptidases were identified in the midguts of two other tick species, *Rhipicephalus appendiculatus *[16] and *Hæmaphysalis longicornis *[17]. Aside from these few reports, little else is known.

In view of the importance of the midgut proteins in understanding the process of blood-meal digestion as well as the fate of microbial infections, we constructed a midgut cDNA library from blood-fed females of the American dog tick, *D. variabilis*. We obtained and analysed 1,679 high-quality sequenced random clones from the PCR-based cDNA library which generated 835 unique transcripts. Putative functional assignments were made using BLAST homologies to other proteins in the National Center for Biotechnology Information (NCBI) nonredundant database (NR) and by comparison with a specialised database containing all Acari protein sequences (ACARI) and the EuKaryotic Orthologous Groups (KOG), Protein families and domains (Pfam) and Self-Monitoring, Analysis, and Reporting Technology (SMART) protein databases. Here we report the 835 unique transcripts assembled from these clones and the putative assignment of 418 of these sequences among 24 different major protein categories.

## Results and discussion

### Library construction

A total of 1,152 plaque phages were sequenced from each of the two constructed cDNA libraries for a total of 2304 5' ESTs. A total of 1,679 high quality sequences, including 771 sequences from the 6-day-fed and 908 sequences from the unfed/2-day-fed libraries, were included in the bioinformatic analysis. Redundant sequences were clustered into related groups using BLASTN and then assembled into contiguous sequences using the CAP3 assembler yielding 835 unique transcripts (labeled DvM 1-DvM 835 representing *D. variabilis *midgut) of which 129 were derived from two or more ESTs (concensus sequence) and 706 were derived from a single EST (singleton). The 835 unique sequences were compared using the program BLASTX, BLASTN, or RPS-BLAST [18] to the NR protein NCBI database, to a custom-prepared ACARI database, the Gene Ontology (GO) database [19], and to the NCBI conserved domains database (CDD) including KOG, PFAM and SMART [20]. The three-frame translations of each sequence were inspected for the presence of a signal peptide using the SignalP server [21]. After removal of vector sequence and poor quality sequences, all remaining sequenced cDNA transcripts produced in this study were submitted to dbEST, a database of expressed sequence tags (EST) on GenBank. The accession numbers for the unfed/2-day-fed cDNA midgut library are EX744988 – EX745928 and 6-day-fed cDNA midgut library accession numbers are EX743967 – EX744987. Transcripts for which a putative amino acid sequence could be deduced and predicted function could be ascertained were submitted to GenBank (EU551603–EU551651).

### Assignment of functional classes

Approximately half (50.2%) of the expressed genes derived from analysis of the cDNA libraries could be assigned to specific functional categories based on significant homologies to functionally assigned proteins found on GenBank. Using best match results to the GO and KOG databases as a guideline, transcripts were assigned to one of 24 classes based on biological function or to a group of "uncharacterised conserved function" or "unknown" class (Table 1). The 24 classes included such biological functions as immunity, metabolism, protein export, protein synthesis and modification machinery, signal transduction, transporter, nuclear regulation, lipid binding and transcription. The majority of transcripts (n = 396, 47%) were classified as unknown due to no match to any of the analysed databases or a non-significant or irrelevant match. The most abundant class, both in terms of the number unique transcripts and total ESTs, was classified as protein synthesis machinery which contains 32 concensus sequences and 67 singletons totaling 655 ESTs. This abundance is due, in part, to DvM 2 which consists of 480 ESTs. DvM 2 is a partial sequence and based on a BLASTN search against a subset mitochondrial gene database, is most related to *Rhipicephalus sanguineus *mitochondrial DNA (AF081829, 4E-056 E-value). The remainder of this class is composed of ribosomal, mitochondrial and other housekeeping genes involved in protein synthesis. The abundance of mitochondrial and ribosomal protein coding genes is not unusual for a transcriptome analysis and illustrates the high degree of redundancy found in the libraries, especially the occurrence of numerous sequences coding for proteins involved in protein synthesis such as ribosomal RNA, e.g. 40S, 60S and other ribosomal genes. The second largest functional class includes proteins involved in energy functions of metabolism (metabolism, energy containing 163 ESTs which generated 23 concensus sequences and 47 singletons) such as NADH dehydrogenase, cytochrome c oxidases, cytochome b, adenosine triphosphate (ATP) synthase and other synthetases (Table 1). As with proteins associated with protein machinery, proteins involved in metabolism, energy, were highly abundant. The abundance of energy facilitating proteins is a feature consistent with the active role of the midgut in cell growth and hemoglobin digestion during blood feeding.

**Table 1:** Major categories of biological functions

**Biological Function**	**ESTs**	**Transcripts**	**Transcripts**		**No. of ESTs**		**ESTs per Transcript**	
			**unfed/2 d fed**	**6 d fed**	**unfed/2 d fed**	**6 d fed**	**unfed/2 d fed**	**6 d fed**
cytoskeletal	17	17	10	7	10	7	1	1
extracellular matrix	11	6	6	1	7	4	1.17	4
immunity	35	7	4	5	10	25	2.50	5.00
lipid binding	4	4	3	1	3	1	1.00	1.00
metabolism, amino acid	36	24	5	21	6	30	1.20	1.43
metabolism, carbohydrate	12	9	3	7	3	9	1.00	1.29
metabolism, energy	163	70	39	44	118	45	3.03	1.02
metabolism, heme	5	2	2	0	5	0	2.50	0.00
metabolism, lipid	17	14	3	12	3	14	1.00	1.17
metabolism, nucleic/nucleotide	1	1	0	1	0	1	0.00	1.00
nuclear regulation	20	11	6	8	9	11	1.50	1.38
proline rich protein	5	5	4	1	4	1	1.00	1.00
peptidase inhibitor	10	9	3	6	3	7	1.00	1.17
proteasome machinery	7	7	3	4	3	4	1.00	1.00
protein export machinery	6	6	1	5	1	5	1.00	1.00
protein modification machinery	47	35	15	21	17	30	1.13	1.43
protein synthesis machinery	655	99	68	49	393	262	5.78	5.35
secreted	112	46	29	22	44	68	1.52	3.09
signal transduction	14	13	8	6	8	6	1.00	1.00
transcription factor	8	7	4	4	4	4	1.00	1.00
transcription machinery	10	10	8	2	8	2	1.00	1.00
transporter	20	15	5	11	8	12	1.60	1.09
transposable element	1	1	0	1	0	1	0.00	1.00
Uncharacterized conserved function	24	21	14	9	15	9	1.07	1.00
Unknown	439	396	212	192	226	213	1.07	1.11

**Total**	1679	835	455	440	908	771	1.40	1.58

Other notable classes of biological function include secreted proteins for which there were 9 concensus sequences and 37 singletons containing a predicted signal peptide, protein modification machinery (5 concensus sequences and 30 singletons) that contains transcripts coding for proteins involved in oxidative stress such as glutathione S-transferase (GST) and glutaredoxins, peptidase inhibitors and amino acid (aa) metabolism which includes cysteine, serine and aspartic peptidases. Included among the transcripts in the cytoskeletal category were two singletons identified as tropomyosin (DvM 201) and troponin (DvM 290). Troponin and tropomyosin together form the tropomyosin protein complex that serves as ca^++ ^binding proteins, especially the calmodulin-like proteins, identified as CLSP (calmodulin-like skin proteins in human skin). These proteins are members of the calmodulin family and troponins. Calmodulin is important as a signaling protein as well as a cellular regulator where it functions as a second messenger, as a regulator of calcium-dependent enzymes, and many other cellular functions. Periodic contraction of the smooth muscles surrounding the midgut is believed to be important during blood feeding.

Comparative analysis of the two cDNA libraries indicated that each library (unfed/2-day vs. 6-day-fed) were evenly represented in the combined analysis with 455 and 440 unique transcripts, respectively (Table 1). The average number of ESTs contributing to each transcript was also similar (1.40 and 1.58). Notable exceptions to this are the categories containing aa metabolism and lipid metabolism transcripts which contain more transcripts from the 6-day-fed midgut library than the unfed/2-day-fed library (Table 1).

### The most abundant transcripts identified from *D. variabilis *midgut transcriptome

Table 2 summarises the 50 most abundant transcritps (3 or more ESTs) identified from the combined cDNA library analysis. The table indicates the number of ESTs contributing to the concensus sequence, the representative EST for each concensus and the best match based on the BLASTX algorithm to the NR database and a customised database (ACARI) including all Acari protein sequences found in GenBank, and the accession number for each match. The most abundant transcript (DvM 2) comprises 480 ESTs associated with mitochondrial DNA. The second most abundant transcript (DvM 4) has an unknown function and, interestingly, appears to be secreted with a predicted signal peptide between cleavage positions 24 and 25 (VLS-QE), based on peptide comparison using the Signal P server [22]. The protein sequence for DvM 4 appears to be full length, based on the presence of a predicted signal peptide and a polyA tail preceeded by a stop codon, and has a molecular weight of 18.3 kDa and an isoelectric point (pI) of 8.50. The third most abundant transcript (DvM 5), comprised of 28 ESTs, is associated with the metabolic lipid (ML) domain protein, important in pathogen recognition and innate immunity. The fourth most abundant transcript (DvM 6) with 22 ESTs with similarity to cytochrome oxidase 1 and the fifth most abundant transcript (DvM 7) respresented by 19 ESTs associated with cytochrome oxidase 3, are both important in mitochondrial metabolic activity. The sixth most abundant transcript represented by 16 ESTs is associated with an unknown salivary protein in *Ixodes scapularis*. The seventh (DvM 1) and eighth (DvM 9) most abundant transcripts (14 and 13 ESTs, respectively) contained no significant matches to the NR or ACARI database which are based on translated BLAST algorithms, but when compared with nucleotide databases such as the mitochondrial-plastid (MIT-PLA) and rRNA subset databases using BLASTN significant homologies were found with mitochondrial DNA species (indicated on Table 2 with an asterisk). The remaining unique transcripts comprised fewer sequences; most (62%) had 5 or less ESTs. Twenty-nine transcripts contained homologies to housekeeping proteins, including 11 concensus sequences with homologies to RNA structure, RNA binding, translation and transcription, and nine transcripts appear to be involved in energy metabolism. When matched against the ACARI and CDD databases, most of the abundant transcripts (66%) showed the highest matches to ticks or other acarines. Three transcripts were recognised as cysteine peptidases (DvM 12, 13, and 42) with close matches to similar peptidases found in *R. haemaphysaloides*, *R. appendiculatus *and *H. longicornis*. One transcript (DvM 10) was recognised as a GST with a very high match to a *D. variabilis *midgut GST; another (DvM 20) was tentatively assigned as a mucin glycoprotein. Eleven transcripts contained unknown sequences with no functional relationship to known proteins or showed conflicting matches with low E-values and, consequently, could not be assigned a function.

**Table 2:** The fifty most abundant transcripts found in the combined unfed/2-d fed and 6-d fed *D. variabilis *midgut libraries.

**Transcript No.**	**No. of EST**	**Representative EST**†	**Best match to NR protein database**	**E-value**	**Genbank**	**Putative Function**
2	480	B04_DVMGL_P10	similar to Plasmodium falciparum	1.3	AAO51426	mitochondrial DNA*
4	36	A09_DVMGS_P8	hypothetical protein [A. thaliana]	0.035	AAG50693	Unknown
5	28	B11_DVMGM_P11	ML domain-containing protein [I. ricinus]	1E-028	AAP84098	ML domain-containing
6	22	DVMG2M-P6_C01	cytochrome c oxidase I [R. sanguineus]	1E-149	NP_008511	cytochrome oxidase 1
7	19	DVMG2L-P12_F09	cytochrome c oxidase III [R. sanguineus]	1E-106	NP_008515	cytochrome oxidase 3
8	16	F04_DVMGL_P10	salivary secreted protein [I. scapularis]	5E-019	AAY66581	salivary secreted protein
1	14	D04_DVMGM_P3				mitochondrial*
9	13	DVMG2S-P4_E04				mitochondrial*
11	9	G06_DVMGM_P11	hypothetical protein [S. dysenteriae]	0.77	YP_405617	mitochondrial*
10	9	C06_DVMGM_P12	glutathione S-transferase [D. variabilis]	2E-082	AAO92279	Glutathione S-transferase
16	8	DVMG2S-P3_B05	ATP synthase F0 subunit 6 [R. sanguineus]	8E-051	NP_008514	ATPase 6
15	8	DVMG2L-P9_H04	NADH dehydrogenase subunit 1 [R. sanguineus]	1E-061	NP_008517	NADH dehydrogenase 1
19	7	DVMG2L-P12_F02	cytochrome b [R. sanguineus]	1E-144	NP_008522	cytochrome b
18	7	DVMG2M-P7_E08	NADH dehydrogenase subunit 3 [R. sang	2E-032	NP_008516	oxidoreductase
17	7	DVMG2S-P3_E03	cytochrome c oxidase subunit II [R. sanguineus]	1E-066	NP_008512	cytochrome oxidase 2
23	6	DVMG2M-P8_D03	hypothetical protein [P. falciparum 3D7]	0.49	CAB39005	Unknown
22	6	DVMG2L-P11_D11	transcription factor [C. neoformans]	0.35	AAW44646	Metallothionein, predicted*
21	6	DVMG2S-P2_B10	Unknown [D. rerio]	0.057	AAI16539	Unknown
20	6	DVMG2L-P11_B01	proline threonine rich protein [Mycobacterium]	2E-009	YP_637621	Mucin characteristics
27	5	DVMG2S-P1_D02	putative salivary secreted peptide [I. pacificus]	4E-008	AAT92122	salivary secreted peptide
26	5	DVMG2M-P8_H10	60S ribosomal protein L13A [M. edulis]	4E-075	ABA46793	60S ribosomal protein L13
25	5	DVMG2L-P9_E07	40S ribosomal protein S12 [D. variabilis]	8E-071	AAP04352	40S ribosomal protein S12
24	5	A02_DVMGM_P11	Phosphatidylinositol 3-4-kinase [T. thermo]	0.60	EAR84499	Unknown
37	4	DVMG2M-P8_A01	similar to Collagen [Mu. musculus]	0.35	XP_992431	Unknown
36	4	DVMG2M-P7_G12	NADH dehydrogenast 4 [R. sanguineus]	8E-062	NP_008519	NADH dehydrogenase 4
33	4	DVMG2L-P10_D10	NADH dehydrogenase 2 [R. sanguineus]	1E-019	NP_008510	NADH dehydrogenase 2
32	4	DVMG2L-P9_G11	Forkhead-associated [Psychrobacter sp.]	0.016	ZP_01272146	Unknown
29	4	E08_DVMGL_P2	guanine nucleotide-binding [I. scapularis]	0.0	AAY66933	Nucleotide binding protein
28	4	B07_DVMGM_P11	Salivary gland secretion [D. melanogaster]	9E-007	NP_523475	Unknown
12	4	B01_DVMGL_P1	cathepsin L-like [H. longicornis]	2E-050	BAA34704	cathepsin L proteinase B
57	3	DVMG2S-P2_H02	predicted protein [M. grisea]	5.1	XP_367470	Unknown
56	3	DVMG2M-P8_B09	ferritin heavy chain-1a [C. rotundicauda]	2E-043	AAW22506	Ferritin
55	3	DVMG2S-P1_G12	similar to 60S ribosomal protein L29 [P. troglod	4E-021	XP_517026	60S ribosomal protein L29
54	3	DVMG2S-P1_A07	ribosomal protein L44 [. pacificus]	3E-057	AAT92163	ribosomal protein L44
53	3	DVMG2S-P4_G09	39S ribosomal protein L12 [S. purpuratus]	1E-037	XP_790377	mitochondrial ribosomal
49	3	DVMG2S-P2_B01	60S ribosomal protein L35 protein [I. pacificus]	9E-057	AAT92193	60S ribosomal protein L35
48	3	DVMG2L-P10_G1	40S ribosomal protein S3a [I. scapularis]	1E-109	AAY66934	40S ribosomal protein S3a
47	3	DVMG2L-P9_F10	ribosomal protein L44 [I. pacificus]	1E-104	AAT01919	ribosomal protein S3
46	3	H12_DVMGL_P2	hypothetical protein [P. troglodytes]	2.1	XP_525349	Unknown
45	3	DVMG2S-P1_A03	40S ribosomal protein S10 [I. scapularis]	6E-032	AAY66831	40S ribosomal protein S10
44	3	C10_DVMGM_P3	ribosomal protein L18a [I. scapularis]	1E-055	AAY66898	ribosomal protein L18a
43	3	DVMG2L-P9_G05	ATP synthase c-subunit [D. variabilis]	5E-067	AAO92282	ATP synthase c-subunit
42	3	C01_DVMGL_P2	midgut cysteine proteinase 1 [R. append]	1E-056	AAO60044	midgut cys proteinase 1
41	3	F05_DVMGL_P1	hypothetical protein [O. sativa]	0.35	NP_914254	Unknown
40	3	DVMG2M-P7_C02	ribosomal protein L12 [I. scapularis]	6E-085	AAY66840	ribosomal protein L12
39	3	A12_DVMGM_P4				unknown
38	3	C09_DVMGL_P1	ribosomal protein S19 [I. scapularis]	2E-040	AAY66936	ribosomal protein S19
34	3	DVMG2M-P6_G07	cytochrome c oxidase I [D. variabilis]	1E-101	AAF61361	cytochrome c oxidase I
30	3	G03_DVMGL_P10	SAICAR synthase [S. purpuratus]	8E-048	XP_801851	SAICAR synthase
13	3	DVMG2L-P11_E05	cathepsin L-like [R. haemaphysaloides]	1E-116	AAQ16117	cathepsin L proteinase A

† Represents clone corresponding to the largest EST. Asterisks (*) indicate assignment of putative function based on a database other than ACARI or NR

### Categories of putative functions involved in tick midgut function

**Figure 1:**
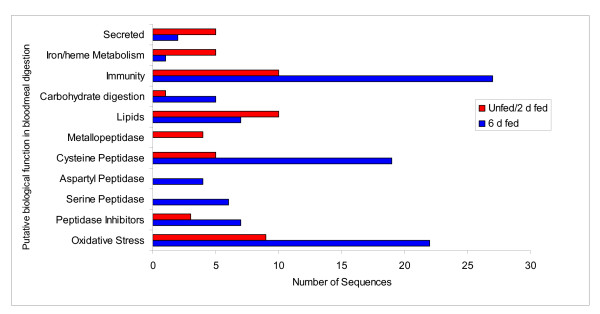
Differential display of proteins associated with midgut function either unfed and 2 days post bloodmeal (unfed/2 d fed) or 6 days post bloodmeal (6 d fed).

Eighty-three transcripts (23 concensus sequences and 57 singletons) were identified as proteins putatively involved directly in blood-meal digestion by *D. variabilis*, based on putative biological function. These include enzymes involved in 1) oxidative stress reduction/antimicrobial activity/detoxification (17 transcripts); 2) peptidase inhibitors (9 transcripts); 3) protein digestion (cysteine-, aspartic-, serine-, and metallo-peptidases) (26 transcripts); 4) cell, protein and lipid binding, including mucins (11 transcripts); 5) carbohydrate digestion (4 transcripts); 6) immunity (8 transcripts); 7) iron/heme metabolism and transport (3 transcripts); and 8) secreted proteins (5 transcriptss). With the exception of metallopeptidases, secreted proteins and transcripts associated with lipid function and iron/heme metabolism and transport, all categories showed putative increased expression during blood feeding (Table 3 and Figure 1). Table 3 summarises the major groups involved in blood feeding and digestion. Each category will be discussed in detail below.

**Table 3:** Categories of proteins potentially involved in blood meal digestion

**Putative Biological Function**	**Total transcripts**	**Total**	**Unfed/2 d fed**	**6 d fed**
			**Number of ESTs**	
Oxidative Stress	17	32	9	23
Peptidase Inhibitors	9	10	3	7
Total Peptidases	26	38	9	29
Serine Peptidase	5	6	0	6
Aspartyl Peptidase	3	4	0	4
Cysteine Peptidase	14	24	5	19
Metallopeptidase	4	4	4	0
Lipids	11	17	10	7
Carbohydrate digestion	4	6	1	5
Immunity	8	37	10	27
Iron/heme Metabolism	3	6	5	1
Secreted	5	7	5	2

### Oxidative stress, oxidase-related antimicrobial activity or detoxification

**Figure 2:**
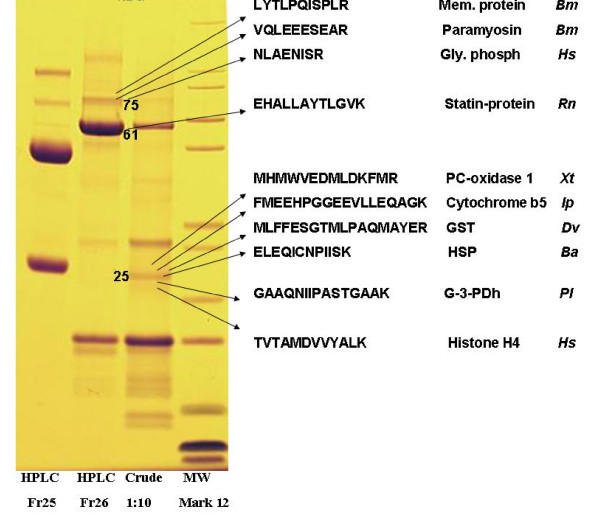
**SDS-PAGE protein gel from a lysate of midguts from 6 d-fed female *Dermacentor variabilis *showing the location of proteins identified by tryptic digestion-mass spectrometry.** Abbreviations: Fr 25, Fr 26 = fractions 25 and 26; Glyphosph = Glycogen phosphorylase; G-3-pdh = glyceraldehydes-3-phosphodehydrogenase; GST = Glutathione S-transferase; HPLC = high performance liquid chromatography; HSP = 70 kD heat shock protein; Mem. protein = Bm86 membrane antigen in *Rhipicephalus (Boophilus) microplus*; MW = molecular weight markers; PC-oxid = prenylcysteine oxidase. *Balanus amphrite *(Ba), *Rhipicephalus (Boophilus) microplus *(Bm), *D. variabilis *(Dv), *Homo sapiens *(Hs), *Ixodes pacificus *(Ip), *Pleocyemata *unclassified (Pl), *Rattus norvegicus *(Rn) and *Xenopus tropicalis *(Xt).

**Figure 3:**
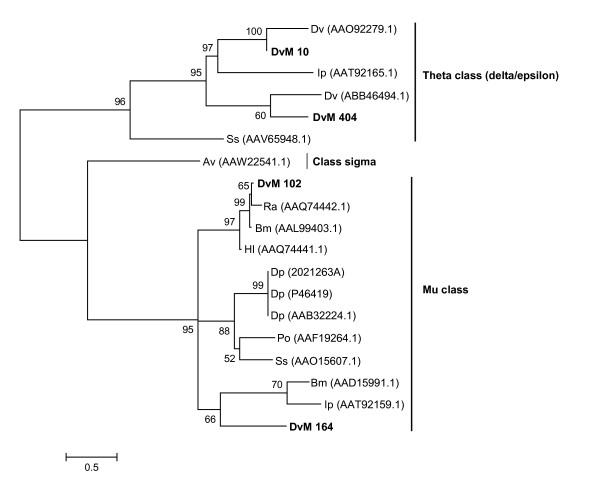
**Analysis of Glutathione-S-transferase (GST) protein family.** Phylogenetic tree based on maximum likelihood analysis of *Dermacentor variabilis *midgut protein and published GST sequences. Transcripts identified in this analysis are in bold. Phylogenetic analysis was conducted on protein alignments using Tree Puzzle version 5.2. Values at the nodes represent calculated internal branch node support (1000 replications). *D. variabilis *(Dv), *Ixodes pacificus *(Ip), *Sarcopte scabiei *(Ss), *Araneus ventricosus *(Av), *Rhipicephalus appendiculatus *(Ra), *R. (Boophilus) microplus *(Bm), *Haemaphysalis longicornis *(Hl), *Dermatophagoides pteronyssinus *(Dp), and *Psoroptes ovis *(Po).

**Figure 4:**
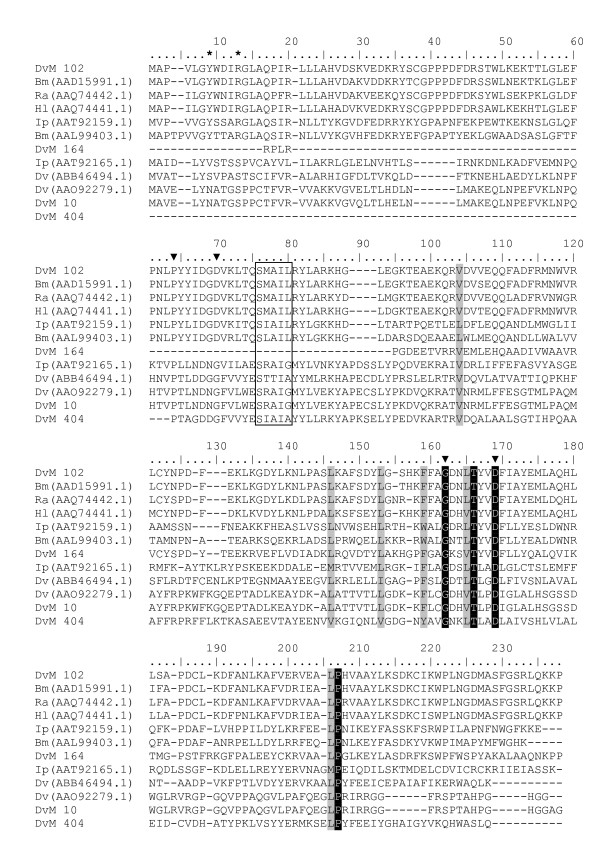
**Multiple sequence alignment of protein sequences identified in a cDNA library of unfed/2 d fed or 6 d fed *D. variabilis *midguts (DvM) and published tick GST sequences found on genbank.** Catalytic residues are indicated by an asterisk and conserved residues involved in the GST fold are indicated by arrowheads. The conserved SMAIL domain is boxed. Shading represents 100% identity (black) or similarity (grey) among the sequences. Alignments were conducted using CLUSTALX. *D. variabilis *(Dv), *Ixodes pacificus *(Ip), *Rhipicephalus appendiculatus *(Ra), *R. (Boophilus) microplus *(Bm), and *Haemaphysalis longicornis *(Hl).

Table 4 lists the 4 concensus sequences and 12 singletons that contained transcripts for proteins expressed in response to oxidative stress, antimicrobial activity or detoxification associated with blood feeding. Most were found in the midguts from 6-day-fed ticks (n = 23, 75%). GST comprised the most common members of this category. Seven putative GST were identified (DvM 10, 102 164, 277, 286, 404 and 695), six of which match GST from other tick species. With but one exception (described below), all were found in the midguts from the 6-day-fed ticks and lacked signal peptides, suggesting they are most likely cytoplasmic proteins. Several of the GST-like transcripts (DvM 10, 102, 286 and 277) are probably not novel because they show excellent matches (99% identity) to GST from *D. variabilis *(AA092279.1) and *Rhipicephalus (Boophilus) microplus *(AAD15991.1). The identity of DvM 10 was also confirmed by tryptic digestion/mass spectrometry (Figure 2). Phylogenetic analysis comparing published GST sequences from non-insect arthropods to those found in this study indicated that DvM 10 and 404 belong to the cytoplasmic GST class Theta (delta/epsilon), whereas DvM 102 and 164 are most closely related to the cytoplasmic Mu class (Figure 3) [23]. Alignment of GST from various tick species supports the phylogenetic placement of our transcripts (Figure 4). Several conserved characteristics also support the phylogenetic placement of these transcripts; DvM 10 (DvM 404, a singleton, was 5' truncated and therefore could not be analysed) contains the hallmark of a Theta class GST with a catalytically essential serine rather then a tyrosine in the N-terminus; the SMAIL/TRAIL conserved motif; and several conserved aa involved in the GST fold [24] (Figure 4). Previous work [24] identified two GST from *D. variabilis *both belonging to the Theta class. This is the first identification of GST from *D. variabilis *belonging to another cytoplasmic GST family, namely the Mu class. The phylogenetic tree supports the functional assignment of these transcripts as GST. GST are known to play an important role in cellular stress responses such as may occur as a result of blood feeding as well as in innate immunity [24-27].

**Table 4:** Transcripts associated with oxidative stress, oxidase-related antimicrobial activity or detoxification

**Transcript**	**Total**	**6 d fed**	**Unfed/2 d fed**	**Sig**	**Putative Function**	**Best match to NR protein database**	**E value**	GenBank*
	**Number of ESTs**							
DvM 453	1	1	0	Cyt	aldehyde dehydrogenase	mitochondrial aldehyde dehydrogenase	2E-047	EU551612
DvM 401	1	1	0	Cyt	glutaredoxin	glutaredoxin [A. aegypti]	3E-017	EU551603
DvM 122	2	0	2	Ind	Glutathione peroxidase	phospholipid-hydroperoxide glutathion	1E-087	EU551610
DvM 10	9	9	0	Cyt	glutathione S-transferase	glutathione S-transferase [D. variablis]	2E-082	EU551642
DvM 102	2	2	0	Cyt	glutathione S-transferase	glutathione S-transferase [B. microplus]	1E-117	EU551607
DvM 164	1	1	0	Cyt	glutathione S-transferase	glutathione S-transferase [H. longicornis]	3E-011	EU551608
DvM 277	1	1	0	Ind	glutathione S-transferase	glutathione S-transferase [D. variablis]	1E-004	
DvM 286	1	1	0	Cyt	glutathione S-transferase	glutathione S-transferase [D. variablis]	2E-026	
DvM 404	1	1	0	Cyt	glutathione S-transferase	glutathione S-transferase [D. variablis]	5E-008	EU551609
DvM 22	6	2	4	Cyt	Metallothionein	ligand-regulated transcription factor	0.35	
DvM 695	1	0	1	SIG	Microsomal GST	MGST 3 [S. purpuratus]	6E-038	EU551606
DvM 258	1	1	0	Cyt	Protein disulfide isomerase	CG5809-PA [A. mellifera]	1E-010	
DvM 565	1	0	1	Ind	quinoid dihydropteridine reductase	quinoid dihydropteridine reductase [G. gallus]	7E-076	
DvM 143	1	1	0	Ind	selenoprotein M precursor	salivary selenoprotein M [I. scapularis]	3E-006	
DvM 199	1	1	0	Ind	superoxide dismutase	superoxide dismutase	3E-041	EU551611
DvM 235	1	1	0	Cyt	Thioredoxin	fed tick salivary protein 3 [I. scapularis]	4E-038	EU551605
DvM 509	1	0	1	Ind	Thioredoxin	hypothetical protein [X. tropicalis]	1E-011	

*Accession number represents transcripts derived from this analysis and submitted to Genbank.

**Figure 5:**
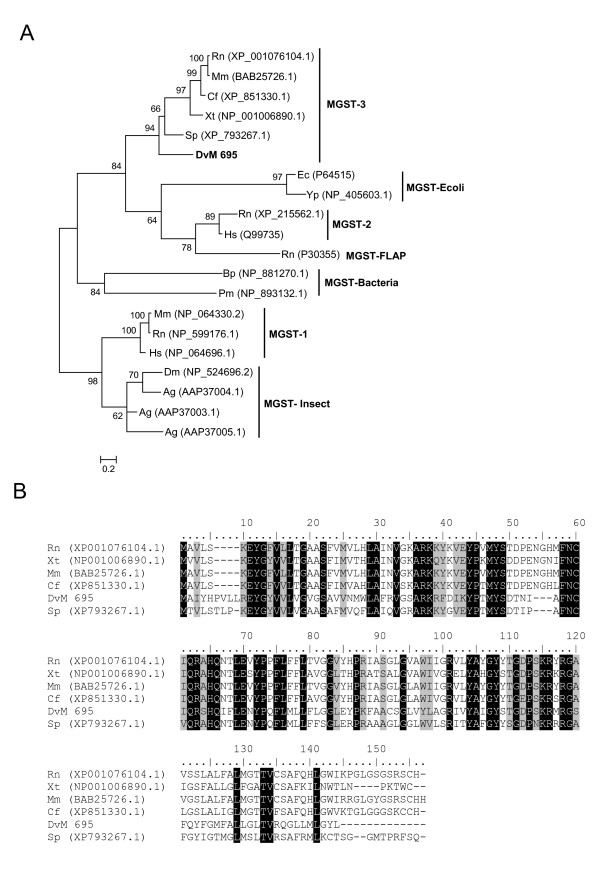
**Analysis of microsomal GST protein sequences.** (A) Phylogenetic tree based on maximum likelihood analysis *Dermacentor variabilis *midgut protein and published microsomal GST sequences. The transcript identified in this analysis is in bold (DvM). Phylogenetic analysis was conducted on protein alignments using Tree Puzzle version 5.2. Values at nodes represent calculated internal branch node support (1000 replications). (B) Multiple sequence alignment (CLUSTALX) of protein sequences identified in a cDNA library of unfed/2 d fed or 6 d fed *D. variabilis *midguts (DvM) and published microsomal GST 3 sequences found on genbank. Shading represents 100% identity (black) or similarity (grey) among the sequences. Alignments were conducted using CLUSTALX. *D. variabilis *(Dv), *Rattus norvegicus *(Nr), *Mus musculus *(Mm), *Canis familiaris *(Cf), *Xenopus tropicalis *(Xt), *Strongylocentrotus purpuratus *(Sp), *Escherichia coli *(Ec), *Yersinia pestis *(Yp), *Homo sapiens *(Hs), *Bordetella pertussis *(Bp), *Prochlorococcus marinus *(Pm), *Drosophila melanogaster *(Dm), *Anopheles gambiae *(Ag).

The last GST, DvM 695, a peptide represented by a single EST found in the unfed/2-day-fed midguts, does not match any tick or acarine species. Rather, it shows a match to a GST from *Strongylocentrotus purpuratus *and appears to be a microsomal GST3 (MGST). The microsomal GST superfamily, termed MAPEG (membrane-associated proteins in eicosanoid and glutathione metabolism) [25], is a glutathione transferase as well as a glutathione-dependent peroxidase. There are six families of MGST including MGST1, 2, and 3, leukotriene C4 synthase (LTC4), 5-lipoxygenase activating protein (FLAP), and prostaglandin E synthase (PGES). Insect MGST are most similar to MGST1 and PGES, yet DvM 695 appears to be most related to MGST3 based on phylogenetic analysis (Figure 5a) and multiple pairwise alignment (Figure 5b). Additionally, the distinctive sequence pattern of known MGST3 proteins, FNC [AIV]QR [AGS]H [AQ] [HQ]-x(2)Ex(2,3)P, was also observed in DvM 695 [26]. *D. variabilis *DvM 695 appears to be the first MGST identified from a tick.

**Figure 6:**
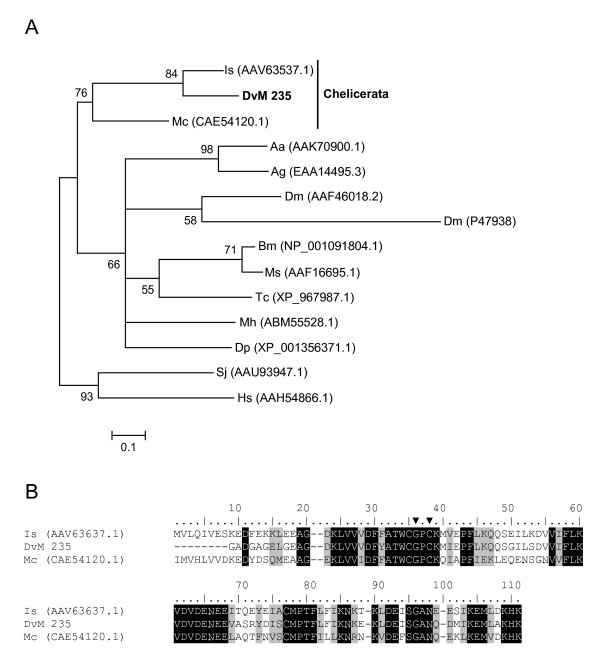
**Analysis of Thioredoxin (TRX) protein family.** (A) Phylogenetic tree based on maximum likelihood analysis of a *Dermacentor variabilis *midgut protein and published TRX sequences. The transcript identified in this analysis is in bold (DvM). Phylogenetic analysis was conducted on protein alignments using Tree Puzzle version 5.2. Values at nodes represent calculated internal branch node support (1000 replications). (B) Multiple sequence alignment (CLUSTALX) of protein sequences identified in a cDNA library of unfed/2 d fed or 6 d fed *D. variabilis *midguts (DvM) and published thiroredoxin-1 sequences found on genbank. Arrowheads indicate the catalytic cysteine motif. Shading represents 100% identity (black) or similarity (grey) among the sequences. Alignments were conducted using CLUSTALX. *D. variabilis *(Dv), *Ixodes scapularis *(Is), *Mesobuthus cyprius *(Mc), *Aedes aegypti *(As), *Anopheles gambiae *(Ag), *Drosophila melanogaster *(Dm), *Bombyx mori *(Bm), *Manduca sexta *(Ms), *Tribolium castaneum *(Tc), *Maconellicoccus hirsutus *(Mh), *Drosophila pseudoobscura *(Dp), *Simulium jonesii *(Sj), and *Homo sapiens *(Hs).

Two transcripts, DvM 235 and 509, each a singleton from the 6-day-fed and unfed/2-day-fed libraries, respectively (Table 4), appear to be antioxidants known as thioredoxins (TRX) which facilitate the reduction of other proteins by cysteine thiol-disulfide exchange (for review see [27]). While thioredoxins are found in nearly all known organisms, only one tick thioredoxin has been deposited in GenBank (AAV63537.1). BLAST analysis of DvM 235 against the NR database revealed a high similarity (E-value 4e-038) to an *I. scapularis *"tick-fed salivary protein 3" (AAV63537.1) identified from a salivary gland cDNA library and was found to be homologous to the thioredoxin-1 protein [28]. This sequence along with DvM 235 was found in the same major clade with other arthropod and non-insect arthropod thioredoxin proteins (Figure 6a) and more specifically, in a sub-clade with a thioredoxin from *I. scapularis*, mentioned previously, and a scorpion thioredoxin (*Mesobuthus cyprius*, CAE54120.1). Additionally, the TRX found from ticks contains the characteristic and essential CXXC motif containing the two vicinal cysteines that enable thioredoxins to reduce other proteins (Figure 6b). In addition to the antioxidant functions of the thioredoxin family, thioredoxin-1, it has been shown that when secreted by antigen presenting cells it is a potent co-stimulator for T-cell activation and growth, and thus is involved in immune functions [29]. DvM 235 appears to be the first thioredoxin found in *D. variabilis *and is putatively assigned to the TRX-1 subfamily. DvM 509 was excluded from subsequent phylogenetic analysis due to N-terminal sequence truncation.

**Figure 7:**
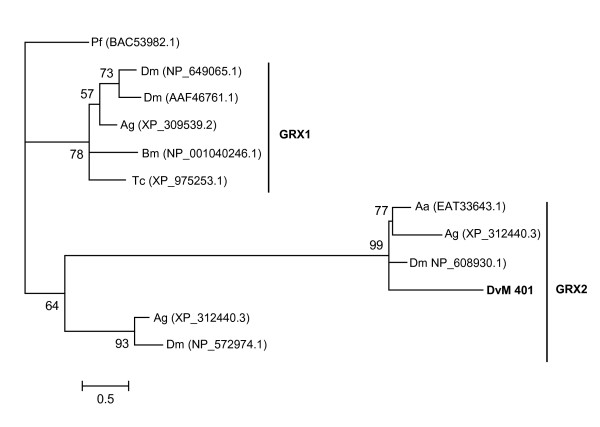
**Analysis of Glutaredoxin (GRX) protein family.** Phylogenetic tree based on maximum likelihood analysis of a *Dermacentor. variabilis *midgut protein and published glutaredoxin (GRX) sequences. The transcript identified in this analysis is in bold (DvM). Phylogenetic analysis was conducted on protein alignments using Tree Puzzle version 5.2. Values at nodes represent calculated internal branch node support (1000 replications). *D. variabilis *(Dv), *Plasmodium falciparum *(Pf), *Drosophila melanogaster *(Dm), *Anopheles gambiae *(Ag), *Bombyx mori *(Bm), *Tribolium castaneum *(Tc), *Aedes aegypti *(Aa).

One transcript (DvM 401), a singleton, found in the 6-day-fed midgut library, was found to be homologous to another antioxidant, glutaredoxin (GRX), which is both structurally and functionally related to TRXs (For review see [27]). In vertebrates, three *Grx *genes have been characterised; GRX1 which is primarily cytoplasmic, GRX2 contain nuclear and mitochondrial variants and GRX5, which is primarily localised in the mitochondria. Some members of the GRX2 group contain a single cysteine residue at the putative active site, whereas like all TRX members, other members of the GRX superfamily have a CXXC cysteine motif. DvM 401 was found in the clade with other GRX2 members (Figure 7). No GRX sequences from acarines were found on GenBank, yet our transcript appears to cluster with other arthropods including an *Aedes ægypti *glutaredoxin (EAT33643.1) to which it is most homologous (Figure 7). Additionally, DvM 401 contains the catalytic site motif, CXXC (data not shown). In ticks, GRX may be involved in trapping reactive oxygen species that would otherwise interact with cellular thiols.

**Figure 8:**
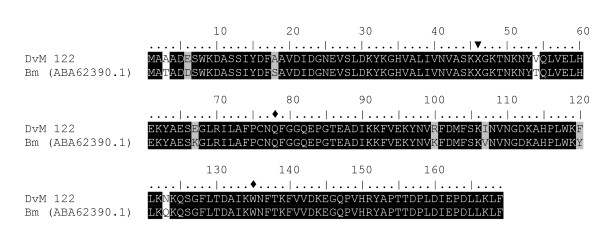
**Analysis of Phospholipid-Hydroperoxide Glutathione Peroxidase (PHGPx) protein family.** Multiple sequence alignment (CLUSTALX) of protein sequences identified in a cDNA library of unfed/2 d fed or 6 d fed *Dermacentor. variabilis *midguts (DvM) and *Rhipicephalus (Boophilus) microplus *(Bm) PHGPx found on genbank. Arrowhead indicates the position of the selenocysteine (X) and the diamond (◆) indicates the active-site residues Gln and Trp that interact with the selenocysteine. Shading represents 100% identity (black) or similarity (grey) among the sequences. Alignments were conducted using CLUSTALX.

Another transcript (DvM 122) found in the oxidative stress related group was found only in the unfed/2-day-fed midgut library of which the deduced aa sequence appears to be homologous to a phospholipid-hydroperoxide glutathione peroxidase (PHGPx) from *Rhipicephalus *(*Boophilus) microplus *(94% identity) (Figure 8). PHGPx, along with glutathione peroxidase (GPx) are selenoenzymes that catalyse the reduction of hydroperoxides in the presence of glutathione [30]. Multiple pairwise alignment of DvM 122 to *R. microplus *PHGPx illustrates the conserved features of this protein such as the codon TAG coding for selenocysteine and the two active site aa (Gln and Trp) which interact with selenocysteine (Figure 8) [31]. Unlike other known PHGPx proteins, DvM 122 as well as the PHGPx from *R. microplus*, does not contain a signal peptide, suggesting that it exists in a cytosolic form. GPx have been identified from salivary gland-specific cDNA libraries of *I. pacificus *(partial sequence, AAT92119.1) and *I. scapularis *(AAK97814.1), and midguts of *I. ricinus *(CAD68003.1) but do not share significant homology to DvM 122 from *D. variabilis*. Aside from *R. microplus*, no other acarine PHGPx has been identified until this study.

**Figure 9:**
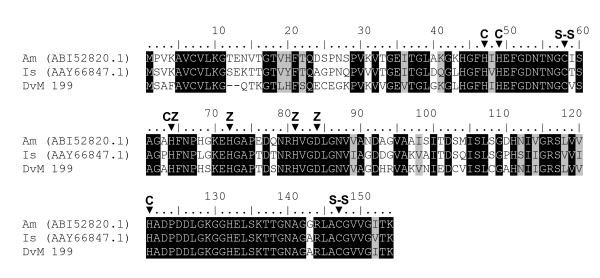
**Analysis of Cu, Zn Superoxide dismutase (SOD) protein family.** Multiple sequence alignment (CLUSTALX) of protein sequences identified in a cDNA library of unfed/2 d fed or 6 d fed *Dermacentor variabilis *midguts (DvM), *Argas monolakensis *(Am) and *Ixodes scapularis *(Is) Cu, Zu SOD. Arrowhead indicates the position of residues that bind either copper (C) or zinc (Z) and the residues that form the disulfide bridges (S-S). Shadding respresents 100% identity (black) or similarity (grey). Alignments were conducted using CLUSTALX.

The tick midgut also shows the presence of enzymes that function as antioxidants by scavenging free radicals known as superoxide dismutase (SOD). One SOD was identified in DvM 199 a singleton expressed in the 6-day-fed midgut library. DvM 199 shared significant identity with Cu, ZnSODs from various species, including a salivary gland specific Cu, ZnSOD from *I. scapularis*. Cu, ZnSODs catalyse superoxide anion into oxygen and hydrogen peroxide by the reduction and oxidation of a metal ion which constitutes the catalytically active redox center [32]. Cu, ZnSOD is important for protection against exogenous oxidative stress by converting superoxide radicals to molecular oxygen. Based on metal binding patterns and signature sequence patterns, DvM 199 appears to be a member of the SOD1 subfamily of superoxide dismutases [33] (Figure 9). Interestingly, Cu, ZnSOD from the bacterium *Hæmophilus ducreyi*, a gram-negative heme obligate coccobacillus, appears to bind heme [34,35]. This suggests that this enzyme, in addition to its antioxidant properties, could function in heme trafficking that would be important in the intracellular tick bloodmeal digestion process.

Transcripts for other oxidative stress peptides include a metallothionen (DvM 22), which may be involved in chelating heavy metals, a selenoprotein (DvM 143), a protein disulfide isomerase (DvM 258), and an aldehyde dehydrogenase (DvM 453) that may function to detoxify aldehydes, such as toxic byproducts resulting from lipid peroxidation (aldehyde detoxification) and may be involved in oxidoreductase activity. The deduced amino acid sequence of DvM 556 shows significant similarity to a quinoid dihydropteridine reductase from *Gallus gallus* (NP_001006566.1, 7E-076 e-value). Quinoid dihydropteridine reductase has oxido-reductase activity involved in tetrahydrobiopterin biosysnthesis and amino acid transport and metabolism.

### Peptidase inhibitors

Table 5 shows 9 unique transcripts, 8 of which are singletons, found to be associated with peptidase inhibitors. Of special interest is the finding that three of the peptidase inhibitors were found only in the unfed/2-day-fed midguts. DvM 626, a putative secreted peptide, showed a match to a similar serpin from the cattle tick, *R. microplus*, known as boophilin, and includes the Kunitz-type trypsin inhibitor domain. DvM 544, a predicted cytoplasmic peptide, also shows the Kunitz-type domain, strongly supporting its role as a serpin despite its poor match in the ACARI and BLAST (NR) databases. DvM 602, another predicted cytoplasmic peptide, is a cystatin with the characteristic cystatin domain. The remaining peptidase inhibitors were found exclusively in the 6-day midguts; four are putative cytoplasmic peptides while the location for the other two could not be determined by the Signal P server.

**Table 5:** Transcripts associated with peptidases inhibitors

**Transcript**	**Total**	**6 d fed**	**Unfed/2 d fed**	**Sig**	**Putative Function**	**Best match to NR protein database**	**E value**	GenBank*
	**Number of ESTs**							
DvM 312	1	1	0	Cyt	serpin – boophilin	secreted protease inhibitor	4E-013	
DvM 626	1	0	1	SIG	serpin – boophilin	boophilin [B. microplus]	7E-031	EU551613
DvM 602	1	0	1	Ind	cytoplasmic cystatin	cytoplasmic cystatin [I. scapularis]	9E-025	EU551615
DvM 544	1	0	1	Cyt	sepin – Kunitz_BPTI	Hypothetical protein [C. elegans]	2.4	EU551614
DvM 226	1	1	0	Ind	secreted cystatin	cystatin-2 precursor [O. moubata]	0.79	EU551616
DvM 334	1	1	0	Cyt	secreted cystatin	secreted cystatin [I. scapularis]	2E-013	EU551617
DvM 422	1	1	0	Cyt	serpin-2	serpin-2 [Ha. longicornis]	3E-022	
DvM 78	2	2	0	Cyt	serpin-3	ENSANGP00000023096 [A. mellifera]	6E-007	
DvM 186	1	1	0	Ind	Zinc-binding protein	GA15490-PA [A. mellifera]	2E-020	

*Accession number represents transcripts derived from this analysis and submitted to Genbank.

**Figure 10:**
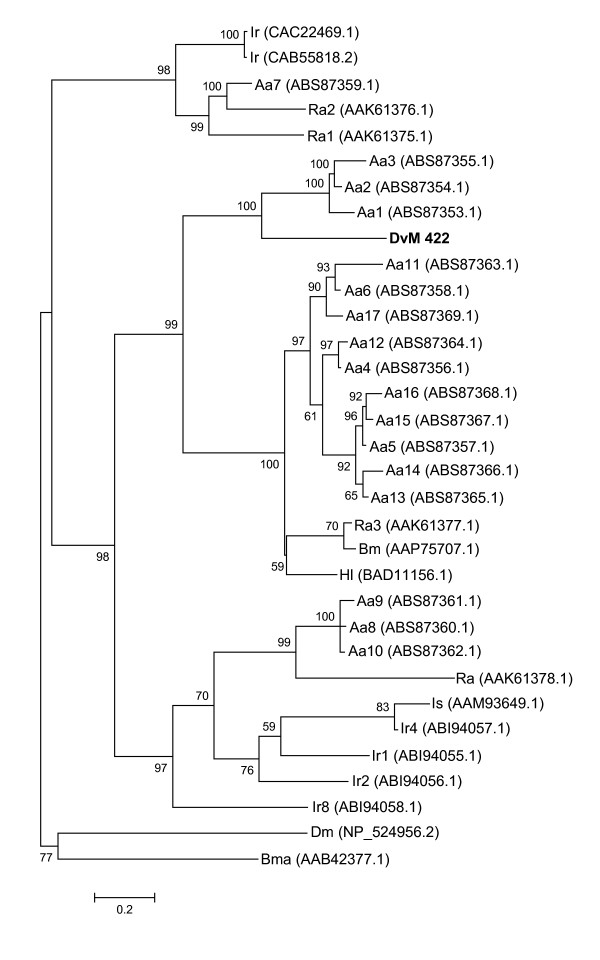
**Analysis of serine peptidase inhibitors (serpins).** Phylogenetic tree based on maximum likelihood analysis *Dermacentor variabilis *midgut protein and published serpin sequences from various tick species including 17 serpins, known as lospin, from *Amblyomma americanum*. The transcript identified in this analysis is in bold (DvM). Phylogenetic analysis was conducted on protein alignments using Tree Puzzle version 5.2. Values at nodes represent calculated internal branch node support (1000 replications). *D. variabilis *(Dv), *Ixodes scapularis *(Is), *Amblyomma americanum *(Aa), *Haemaphysalis longicornis *(Hl), *Rhipicephalus appendiculatus *(Ra), *I. ricinus *(Ir), *Drosophila melanogaster *(Dm), *R. (Boophilus) microplus *(Bm), *Brugia malayi *(Bma).

Two transcripts contained significant matches to serine peptidase inhibitors known as serpins, DvM 422 with 1 EST and 78 with two ESTs, all expressed in 6-day-fed midguts (Table 5). Serpins are important regulators of serine peptidases involved in inflammation, blood coagulation, fibrinolysis and complement activation [36]. Serine peptidase inhibitors include members of the Kazal, Kunitz, α-macroglobin and serpin families. Serine peptidase inhibitors function in insect hemolymph for protection from infection by pathogens or parasites [37]. DvM 422 contains a full-length sequence and is most closely related to Lospin 1, 2, and 3 from *Amblyomma americanum *[38] that are three of 17 serpins found in the Lone Star tick (Figure 10). In *A. americanum *serpins are expressed ubiquitously in the midgut, salivary glands and ovaries with Lospin 1, 2, and 3 having the greatest expression in the midgut [38]. Serpins contain a conserved domain (cd00172) and a reactive center loop, [GREV]-[FRADHP], which is conserved among ticks including our transcript (data not shown). DvM 78 is 5' truncated yet it contained a significant match to a serpin from *H. longicornis *(BAD11156).

**Figure 11:**
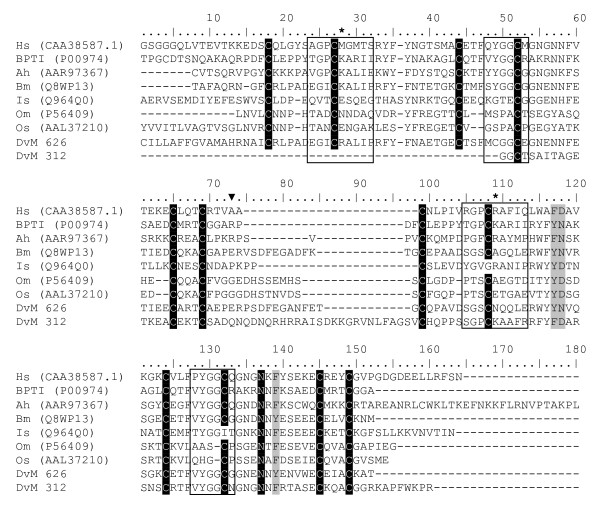
**Analysis of Boophilin-like peptidase inhibitors.** Multiple sequence alignment (CLUSTALX) of protein sequences identified in a cDNA library of unfed/2 d fed or 6 d fed *Dermacentor variabilis *midguts (DvM) and thrombin in hibitor-like proteins found on genbank. Black shading indicates identity and grey shading indicates similarity. Boxed area indicates the amino acids that constitute the peptidase recognition loop. * P1 site, the primary recognition residue. Arrowhead indicates the start of the second Kunitz domain. Shading represents 100% identity (black) or similarity (grey) among the sequences. Alignments were conducted using CLUSTALX. *D. variabilis *(Dv), *Homo sapiens *alpha-1-microglobulin bikunin (Hs), Pancreatic trypsin inhibitor precursor (BPTI), *Amblyomma hebraeum *(Ah), *Rhipicephalus (Boophilus) microplus *(Bm), *Ixodes scapularis *(Is), *Ornithodoros moubata *(Om), *O. savignyi *(Os).

Two singletons (DvM 312 and 626) contained a significant match to a protein named boophilin identified from the cattle tick, *R. microplus *(CAC82583). Although not definitively characterised, boophilin appears to be related to a thrombin inhibitor [39]. Similar to boophilin, both transcripts contain two conserved Kunitz domains (cd00109) and are closely related to a conserved domain for BPTI (bovine pancreatic trypsin inhibitor)/Kunitz family of serine peptidase inhibitors. This domain contains a characteristic disulfide-rich alpha+beta fold as well as a trypsin interaction site ([PCRAMXS]). DvM 312 and 626 contain two Kunitz domains and 6 cysteine residues that together create three disulfide bridges, although DvM 312 is 5' truncated, therefore the first three cysteines of the first domain are absent (Figure 11). A thrombin inhibitor has been identified and functionally characterised from one hard tick, *Amblyomma hebræum *(Amblin, AAR97367) [40] and two soft tick, *Ornithodoros moubata *(Ornithodorin, P56409) [41] and *O. savignyi*, (Savignin, AAL37210) [42]. The prototype for this family, BPTI, contains two recognition loops, the first of which contains a single aa (Lys15) that is the principle determinant of inhibitor specificity. Polymorphisms in these loops contribute to individual species specificity; BPTI inhibits trypsins and boophilin, ambilin and ornithodorin inhibit thrombin. The exact mechanism through which thrombin is inhibited is not understood for hard ticks. The binding appears to be novel in soft ticks, such that neither of the reactive site loops contact the peptidase in the Ornithodorin-thrombin complex; rather, the interaction is through the N-terminal residues [41,42]. It is unknown if the two proteins identified from *D. variabilis *are, in fact, thrombin inhibitors and, if so, if they bind in the same manner as Ornithodorin. Preliminary examination of the sequences obtained from *D. variabilis *suggests an alternative method.

**Figure 12:**
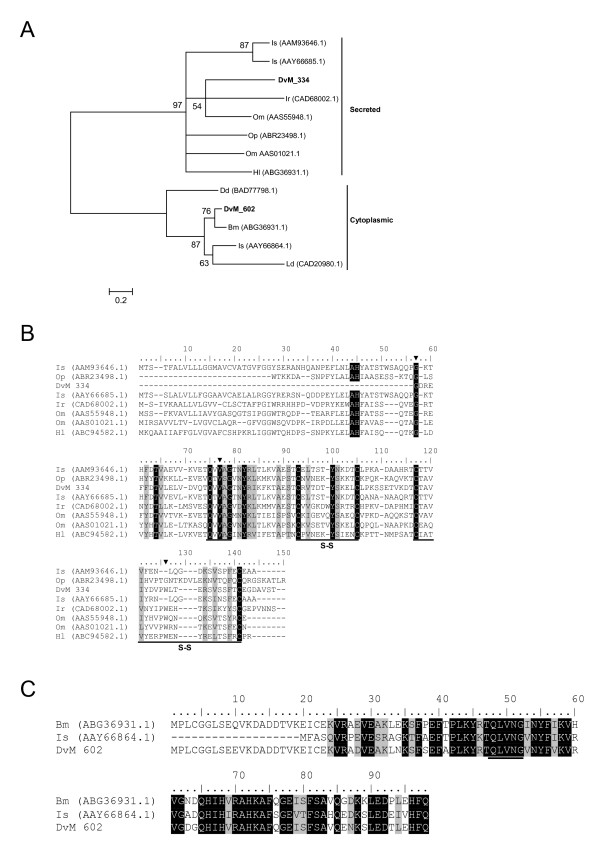
**Analysis of Cystatin a family of cystine peptidases.** (A) Phylogenetic tree based on maximum likelihood analysis of a *Dermacentor variabilis *midgut protein and published secreted and cytoplasmic cystatin sequences. The transcripts identified in this analysis are in bold (DvM). Phylogenetic analysis was conducted on protein alignments using Tree Puzzle version 5.2. Values at nodes represent calculated internal branch node support (1000 replications). Multiple sequence alignment (CLUSTALX) of protein sequences identified in a cDNA library of unfed/2 d fed or 6 d fed *D. variabilis *midguts (DvM) and published secreted (B) and cytoplasmic (C) cystatin sequences found on Genbank. Arrowheads indicate putative papain binding domains. S-S indicates cysteines involved in disulfide bridge formation. The underlined amino acids highlights the conserved QNVLG domain. Shading represents 100% identity (black) or similarity (grey) among the sequences. Alignments were conducted using CLUSTALX.*D. variabilis *(Dv), *Ixodes scapularis *(Is), *I. ricinus *(Ir), *Ornithodoros moubata *(Om), *Haemaphysalis longicornis *(Hl), *Dictyostelium discoideum *(Dd), and *Lepidoglyphus destructor *(Ld).

Deduced aa sequences of three singletons, DvM 226, 334, and 602 shared identity to cysteine peptidase known as cystatins. All three consensus sequences were truncated at the N-terminus; thus complete sequences could not be evaluated. DvM 226 appears to be identical to DvM 334, yet is significantly more truncated and thus is not represented in the alignments or phylogenetic analysis. Based on multiple pairwise alignment and phylogenetic analysis, there appears to be two cytoplasmic and one secreted cystatin found in the cDNA libraries from *D. variabilis *midguts (Figure 12). DvM 602 appears to be most related to *R. microplus *putative intracellular cystatin (ABG36931.1) and *I. scapularis *cytoplasmic cystatin (AAY66864). Although the aminoterminal region is missing, based on phylogenetic similarity we can presume that DvM 602 is a cytoplasmic cystatin (Figure 12a, c). Unlike secreted cystatins, intracellular cystatins do not present with predicted disulfide bonds which is observed among our putative cytoplasmic transcripts (Figure 12c). DvM 334 appears to be a secreted cystatin based on phylogenetic analysis (Figure 12a, c). Grunclova et al. [43] described two secreted cystatins from the soft tick *O. moubata*. DvM 334 (and DvM 226) is most similar to the two secreted cystatins from the soft tick as well as known secreted cystatins from *I. scapularis, O. parkeri*, *I. ricinus *and *H. longicornis*. Although no signal peptide is present, due to 5' truncation we have tentatively identified this transcript as a secreted cystatin. DvM 334 conforms with other known cystatins [44] such that the molecule has three papain-binding areas and four conserved cysteines that create two disulfide bridges (Figure 12b). The three papain-binding areas are thought to create a wedge-shaped binding site involved in reversible binding in the active site of cystatin peptidase of the papain family [44]. Grunclova et al. [43] found that the gut specific *O. moubata *and midgut *I. ricinus *cystatins shared the third papain-binding site, characterised by a PW hairpin loop. This was not seen among salivary gland *I. scapularis *cystatins but we find this motif in the midgut-specific *D. variabilis *DvM 334 (Figure 12b). In all cystatins from *D. variabilis*, the QNVLG or QVVAG domain is conserved as in other cystatins (Figure 12a, b). Cystatins are important in disrupting the activity of cysteine peptidases, enzymes that are essential for several pathogenic parasites and bacteria. Thus, cystatins not only have the capacity to regulate normal biological processes but may also participate in the defense against microbial infections [45].

### Peptidases

Peptidases comprise the largest number of putative expressed genes associated with bloodmeal digestion. Twenty-six transcripts consisting of 38 ESTs were divided among four major groups of pepidases found in the midgut of *D. variabilis*; serine, aspartic, cysteine and metallo (Table 6).

**Table 6:** Transcripts associated with peptidases

**Transcript**	**Total**	**6 d fed**	**Unfed/2 d fed**	**Sig**	**Putative Function**	**Best match to NR protein database**	**E value**	GenBank*
	**Number of ESTs**							
**Serine Peptidase**								
DvM 60	2	2	0	Cyt	serine proteinase-1	serine proteinase-1 [R. appendiculatus]	5E-025	EU551615
DvM 283	1	1	0	Ind	serine carboxypeptidase	CG3344-PA [T. castaneum]	2E-010	
DvM 394	1	1	0	Ind	serine arboxypeptidases	carboxypeptidase [T. castaneum]	3E-004	
DvM 330	1	1	0	Ind	serine protease	serine protease-like protein [O. moubata]	5E-006	EU551620
DvM 210	1	1	0	Ind	serine proteinase 2	serine proteinase 2 [H. longicornis]	3E-047	EU551619
								
**Aspartic Peptidase**								
DvM 249	1	1	0	Ind	aspartic protease	aspartic protease [H. longicornis]	3E-011	EU551622
DvM 254	1	1	0	SIG	Cathepsin D	aspartic protease [H. longicornis]	2E-020	EU551623
DvM 108	2	2	0	Cyt	aspartic proteinase	aspartic protease [H. longicornis]	5E-040	EU551621
								
**Cysteine Peptidase**								
DvM 314	1	1	0	SIG	Cathepsin B peptidase	cathepsin B precursor [A. ventricosus]	9E-087	EU551624
DvM 364	1	1	0	Anch	Cathepsin B peptidase	cathepsin B endopeptidase [shisto	1E-058	EU551628
DvM 42	3	2	1	Ind	Cathepsin B peptidase	cysteine proteinase 1 [R. appendiculatus]	1E-056	EU551629
DvM 13	3	2	1	SIG	cathepsin L peptidase A	cathepsin L [R. haemaphysaloides]	1E-116	
DvM 14	1	1	0	Cyt	cathepsin L peptidase A	cathepsin L [R. haemaphysaloides]	3E-038	EU551631
DvM 12	4	4	0	Cyt	cathepsin L peptidase B	cathepsin L [H. longicornis]	2E-050	EU551630
DvM 247	1	1	0	Ind	cathepsin L peptidase B	homologue of Sarcophaga 26,29kDa	2E-011	
DvM 104	2	1	1	Cyt	cathepsin L peptidase B	cysteine proteinase 2 [R. appendiculatus]	8E-055	EU551632
DvM 542	1	0	1	Ind	cysteine peptidase 2 CL	cysteine proteinase 2 [R. appendiculatus]	3E-020	EU551633
DvM 287	1	1	0	Cyt	cysteine peptidase 5 CL	cysteine proteinase 5 [R. appendiculatus]	9E-023	
DvM 395	1	1	0	Cyt	cysteine peptidase 5 CL	cysteine proteinase 5 [R. appendiculatus]	1E-023	
DvM 62	2	2	0	Cyt	legumain-like protease	legumain protease precursor [I. ricinus]	0.001	EU551625
DvM 96	2	2	0	Cyt	legumain-like protease	legumain protease precursor [I. ricinus]	4E-035	EU551626
DvM 694	1	0	1	Anch	legumain-like protease	Legumain protease precursor [I. ricinus]	1E-110	EU551627
								
**Metallopeptidase**								
DvM 594	1	0	1	Sig	metallopeptidase	Is6 [Ixodes scapularis]	7.E-22	
DvM 732	1	0	1	Ind	metallopeptidase	Proliferation-associated 2G4	1.E-12	
DvM 675	1	0	1	Cyt	mitochond processing	MGC78954 protein [X. laevis]	8E-054	EU551636
DvM 806	1	0	1	Cyt	metallopeptidase	Membrane-type 1 matrix metalloproteinase	1.E-09	

*Accession number represents transcripts derived from this analysis and submitted to Genbank.

#### Serine peptidases

**Figure 13:**
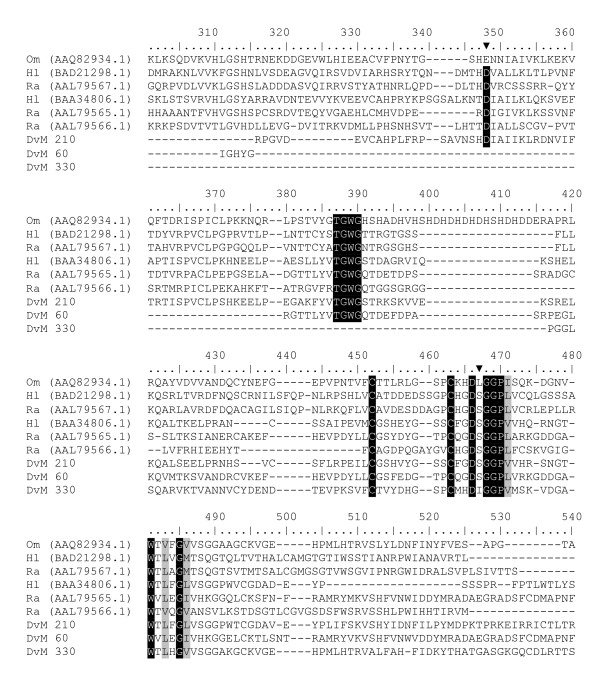
**Analysis of serine peptidases.** Multiple sequence alignment (CLUSTALX) of protein sequences identified in a cDNA library of unfed/2 d fed or 6 d fed *Dermacentor variabilis *midguts (DvM) and tick serine peptidases found on genbank. Arrowheads conserved amino acids involved in the catalytic triad. Shading represents 100% identity (black) or similarity (grey) among the sequences. Alignments were conducted using CLUSTALX. *D. variabilis *(Dv), *Ornithodoros moubata *(Om), *Haemaphysalis longicornis *(Hl), *Rhipicephalus appendiculatus *(Ra).

Trypsins and chymotrypsins are the most extensively studied digestive serine peptidases in haematophagous insects. Five transcripts were found that matched serine peptidases, all found only in the 6-day-fed midguts. Three transcripts (DvM 60, 210, and 330) showed high levels of similarity to serine peptidases from other tick species. All three transcripts were truncated at the aminoterminus, thus no secretion potential could be evaluated. Miyoshi *et al *[17] described a serine peptidase from *H. longicornis *(AB127388) that contained the three conserved catalytic aa typical for chymotrypsin-like proteins (His-Asp-Ser). Alignment of DvM 60, 210 and 330 with *H. longicornis *and other tick serine peptidases from GenBank revealed the extent of the 5' truncation of the three *D. variabilis *transcripts, yet for DvM 210 the second (D) and third (S) catalytic aa in the trypsin catalytic triad were conserved (Figure 13). Additionally, although we were only able to sequence the 3' fragments of the protein, DvM 60 contains the conserved serine whereas DvM 330 contained an isoleucine in place of the serine. As seen in other hemotophogus arthropods [46], it may be possible that some of the serine peptidases found in the tick midgut may be associated with immunity rather then digestive function.

Two transcripts (DvM 283 and 394) were found to be most closely related to serine carboxypeptidases. Serine carboxypeptidases (SCP) are proteolytic enzymes that exploit serine in their catalytic activity, as with chymotrypsins, SCP have a catalytic triad of serine, aspartate and histidine (SDH) whereas chymotrypsins have the HDS triad, which catalyses hydrolysis of C-terminal residues in peptides and proteins as acidic pH. A SCP from *H. longicornis *(BAF64246.1) was described by Motobu et al. [47], which contained the catalytic triad at positions 178, 450 and 397. Alignment of DvM 238 with a SCP from *H. longicornis*, a salivary SCP from *I. scapularis *and a SCP from the jewel wasp, *Nasonia vitripennis *illustrates that, although truncated at the N-terminus, DvM 238 contains the second (Asp) and third (His) aa of the triad (data not shown). Without further analysis of the full-gene product, it is impossible to determine whether the first catalytic aa is conserved. Based on identity with *N. vitripennis *(E-value 7e-24), it seems probable that DvM 283 and DvM 394 are midgut serine carboxypeptidases.

#### Aspartic peptidases

Three transcripts putatively assigned as aspartic peptidases were identified in the midgut libraries. Aspartic peptidases are a family of proteins that include pepsins, cathepsin D, cathepsin E and renins, and are believed to be important in hemoglobin proteolysis [48]. These enzymes are involved in degradation of intracellular and endocytosed proteins and thus have been implicated in blood meal digestion in some ectoparasites, such as mites [49] and ticks [46]. Aspartic peptidases have also been implicated in hemoglobin (Hb) proteolysis [50-52]. Among ticks, an aspartic peptidase named BYC from *R. microplus *appears to be involved in vitellin (yolk protein) digestion as well as heme binding [53]. We identified transcripts similar to an aspartic peptidase, cathepsin D (Table 6).

**Figure 14:**
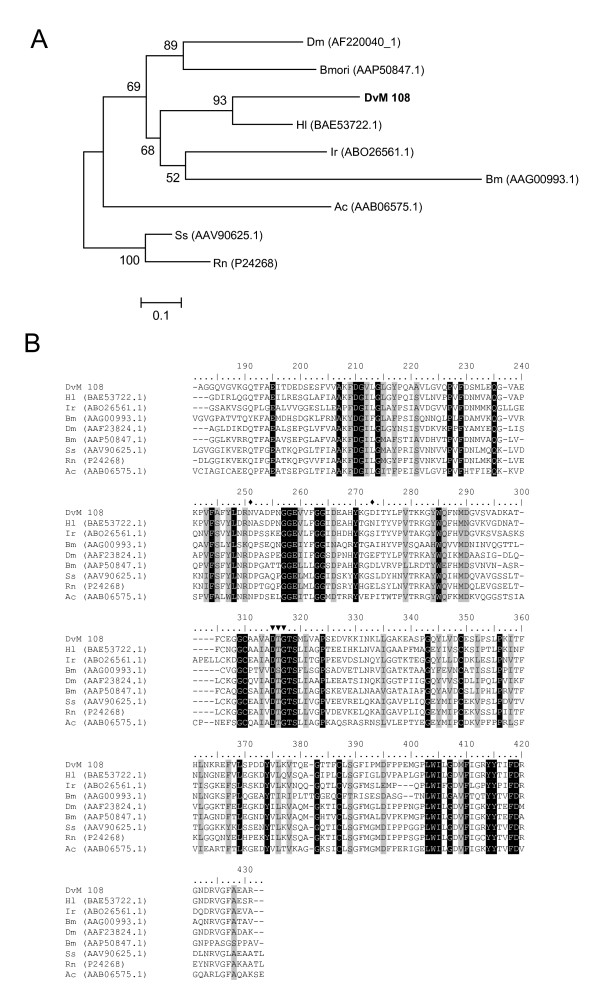
**Analysis of aspartic peptidases, Cathepsin D.** (A) Phylogenetic tree based on maximum likelihood analysis of a *Dermacentor variabilis *midgut protein and published sequences. The transcripts identified in this analysis are in bold (DvM). Phylogenetic analysis was conducted on protein alignments using Tree Puzzle version 5.2. Values at nodes represent calculated internal branch node support (1000 replications). (B) Multiple sequence alignment (CLUSTALX) of protein sequences identified in a cDNA library of unfed/2 d fed or 6 d fed *D. variabilis *midguts (DvM) and published sequences found on Genbank. Arrowheads indicate conserved aspartic catalytic sites and diamonds indicate putative glycosylation residues (nomenclature derived from Boldaatar *et al. *(49). Shading represents 100% identity (black) or similarity (grey) among the sequences. Alignments were conducted using CLUSTALX. *D. variabilis *(Dv), *Drosophila melanogaster *(Dm), *Bombyx mori *(Bmori), *Haemaphysalis longicornis *(Hl), *Ixodes ricnius *(Ir), and *Rhipicephalus (Boophilus) microplus *(Bm), *Ancylostoma caninum *(Ac), Sus scrofa (Ss), *Rattus norvegicus *(Rn).

Three transcripts (DvM 108, 249, and 254) contained significant matches to a peptidase in *H. longicornis *named longepsin (BAE53722.1), which is known to cleave hemoglobin. All three transcripts were found only in the midguts from 6-day-fed ticks; no clones were found in unfed or 2-day-fed ticks. DvM 249 and 254 were extensively 5' truncated, thus were removed from phylogenetic analysis (Figure 14a). DvM 108 and 249 are probably aspartic peptidases since they have an aspartic acid domain, i.e., Asp-Thr-Gly (DTG) catalytic site motif also found in *H. longicornis *[54] and several other cathepsin D peptidases necessary for its enzymatic activity (Figure 14b). This enzyme is typically found in lysosomes. Boldbaatar et al. [54] suggest that in *H. longicornis*, this enzyme "plays an integral role in the proteolysis of erythrocyte Hb obtained from a host blood meal." The occurrence of a similar aspartic peptidase in the *D. variabilis *midgut suggests that it also is likely associated with hemoglobin digestion. Phylogenetic analysis revealed that DvM 108 is most closely related to *H. longicornis *aspartic peptidase and is found in the same sub-clade as other tick cathepsin-D proteins (*I. ricinus *and *R. microplus*), and is also within a major clade containing other arthropod cathepsins (Figure 14a).

#### Cysteine peptidases

**Figure 15:**
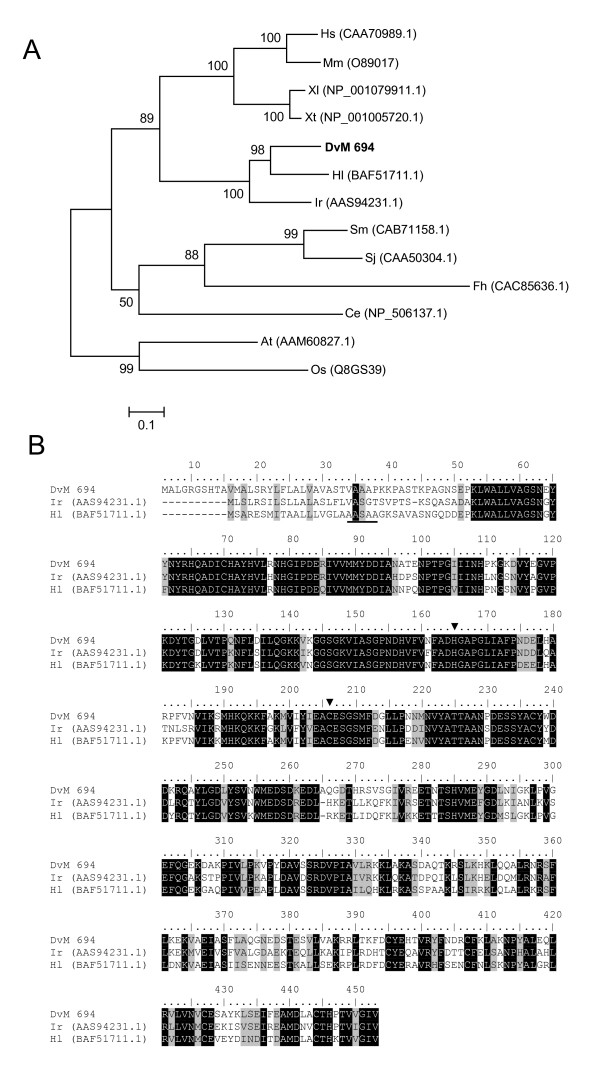
**Analysis of aspartic endopeptidases (AE), a legumain-like family of proteins.** (A) Phylogenetic tree based on maximum likelihood analysis of a *Dermacentor variabilis *midgut protein and published legumain-like sequences. The transcript identified in this analysis is in bold (DvM). Phylogenetic analysis was conducted on protein alignments using Tree Puzzle version 5.2. Values at nodes represent calculated internal branch node support (1000 replications). (B) Multiple sequence alignment (CLUSTALX) of protein sequences identified in a cDNA library of unfed/2 d fed or 6 d fed *D. variabilis *midguts (DvM) and published sequences from other ticks found on Genbank. Arrowheads indicate conserved His and Cys residues forming the catalytic dyad of AE. Underlined amino acids represent the predicted cleavage position of the signal peptide. Shading represents 100% identity (black) or similarity (grey) among the sequences. Alignments were conducted using CLUSTALX. *D. variabilis *(Dv), *Homo sapiens *(Hs), *Mus musculus *(Mm), *Xenopus laevis *(Xl), *Xenopus tropicalis *(Xt), *Haemaphysalis longicornis *(Hl), *Ixodes ricnius *(Ir), *Schistosoma mansoni *(Sm), *Schistosoma japonicum *(Sj), *Fasciola hepatica *(Fh), *Caenorhabditis elegans *(Ce), *Arabidopsis thaliana *(At), and *Oryza sativa *(Os).

**Figure 16:**
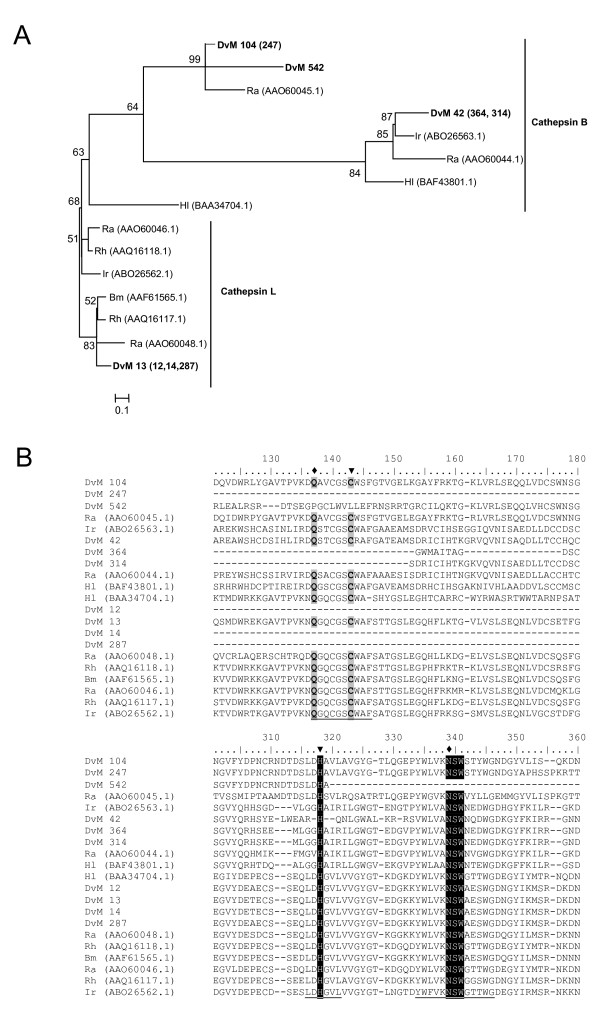
**Analysis of cysteine peptidases.** (A) Phylogenetic tree based on maximum likelihood analysis of a *Dermacentor variabilis *midgut protein and published tick Cathepsin L and B-like peptidase sequences. The transcript identified in this analysis is in bold (DvM). Phylogenetic analysis was conducted on protein alignments using Tree Puzzle version 5.2. Values at nodes represent calculated internal branch node support (1000 replications). (B) Multiple sequence alignment (CLUSTALX) of protein sequences identified in a cDNA library of unfed/2 d fed or 6 d fed *D. variabilis *midguts (DvM) and published sequences from other ticks found on Genbank. Arrowheads indicate catalytic residues. Diamonds represent predicted amino acids involved in catalysis. Shading represents 100% identity (black) or similarity (grey) among the sequences. Alignments were conducted using CLUSTALX. *D. variabilis *(Dv), *Ixodes ricinus *(Ir), *Haemaphysalis longicornis *(Hl), *Rhipicephalus (Boophilus) microplus *(Bm), *R. appendiculatus *(Ra), *R. haemaphysaloides *(Rh).

Fourteen transcripts were found, comprising either multiple or single sequences, that matched cysteine peptidases in the ACARI and/or NR databases and at least one of the CDD databases. Of the 24 total EST containing homology to cysteine peptidases, 19 ESTs were found in the 6-day-fed midguts, whereas only 5 ESTs were found in the 2-day-fed library, suggesting that the expression of this protein family is induced during feeding (Table 6). Phylogenetic analysis revealed three major groups of cysteine peptidases identified among the two cDNA libraries; legumain-like, cathepsin B-like and cathepsin L-like (Figure 15a and Figure 16a).

DvM 62, 96, and 694 share significant sequence similarity to a legumain-like protease from *I. ricinus*. Legumain-like proteases are members of the cystein peptidase family (Clan CD, Family C13) and act specifically as asparaginyl endopeptidases with strict cleavage specificity. Asparaginyl endopeptidases (AE) appear to process other proteins and have been found to be involved in peptide processing before MHC-II loading [55], protein processing during seed germination, and, possibly the most relevant to the hematophagous behavior of ticks, AE have been shown to activate cathepsin zymogens in the gut of blood feeding helminthes such as *Schistosoma mansoni *– allowing Hb digestion [56]. Recently, AE orthologs have been described in two hard ticks, *I. ricinus *[50] and *H. longicornis *[51]. Each has been shown to function as hemoglobinase. Of the three transcripts found in the *D. variabilis *library, two (DvM 62 and 96) appear to be 5' truncated thus were removed from phylogenetic analysis. The third transcript, DvM 694, appears to be full length and contains a signal peptide (VAA-AP), indicating its likely secretion from the cell. DvM 694 shares 69% identity with *I. ricinus *(AAS94231.1) and 74% identity with *H. longicornis *(BAF51711.1) tick legumain and is found in a clade with other known tick-derived AE (Figure 15a). Alignment of the three known tick AE is shown in Figure 15b. The residues His and Cys, involved in forming the catalytic dyad of AE, are conserved among all three ticks including the transcript identified in this analysis (Figure 15b). DvM 62 and 96 each contain two ESTs found in the six days post-attachment midguts, whereas DvM 694 is a singleton expressed in the midgut of unfed or 2-day-fed ticks. The three transcripts share 97% aa similarity with only five aa differences between them; yet without the entire sequence it is impossible to know if the three share a similar function. Potential differences in the N-terminus could explain why the two truncated transcripts are found in the midguts of the 6-day-fed ticks

DvM 314, 364 and 42 shared significant identities with cathepsin-B endopeptidases, while DvM 12, 13, 14, 247, and 104 were most similar to cathespsin-L cysteine peptidases. DvM 542, 287 and 395 were similar to midgut cysteine peptidases from *R. appendiculatus*. With the exception of DvM 13, all other transcripts were 5' truncated. Based on homology to known proteins, DvM 13 contains a predicted signal cleavage site (TTA-AS) suggesting secretion from the cell (data not shown). Multiple sequence alignment of tick cysteine peptidases indicated that the aa involved in the catalytic dyad (Cys and His), which are present in all papain-like cysteine peptidases, were conserved among the tick species, with the exception of DvM 542, which did not contain Cys (Figure 16b). It has been shown that two other residues play an important role in catalysis, a Gln preceding the catalytic Cys, believed to help in the formation of the oxyanion hole, and an Asn residue which orients the imidazolium ring of the catalytic His. As with the catalytic dyad, Gln and Cys were also conserved among the *D. variabilis *transcripts. Although there was little homology among the entire length of sequences, the residues surrounding the catalytic aa were equally conserved (Figure 16b).

#### Metallopeptidases

Four putative metallopeptidases were found among the transcripts. All were found only in the midguts from unfed/2-day-fed ticks. Results from the NR and GO database identified a PA2G4-like (Proliferation Associated Protein 2G4) conserved domain in DvM 732, whereas a match to a metallopeptidase was identified based on a search of the KOG database (E-value 3e-012). PA2G4 is related to an aminopeptidase M which is implicated in cell-cycle control. One metallopeptidase, DvM 594, was homologous to an *I. scapularis *salivary gland secreted protein, Is6 (AAO85923.1), based on searches of the NR and ACARI databases. DvM 806, related to a metallopeptidase, is homologous to a membrane-type 1 matrix metalloproteinase cytoplasmic tail binding protein-1 (E-value 3e-009) based on comparison to the NR database. A final metallopeptidase, DvM 675, appears to have mitochondrial processing peptidase activity and is related enzymes in the insulinase super family (Clan ME, Family M16) based on the best match to the KOG database (E-value 2e-062). In all four examples, the protein appears to be truncated, thus functional active sites indicative of metallopeptidases remain to be confirmed.

### Carbohydrate digestion/Hydrolases

Table 7 lists transcripts for proteins probably associated with carbohydrate digestion, based on the presence of glycosyl hydrolase, beta-galactosidase and tetrahydrofolate dehydrogenase domains. DvM 107, with 2 ESTs, was represented in both the 6-day and the unfed/2-day-fed midguts; the others were found only in the 6-day-fed midguts. Three of the four transcripts, DvM 63, 107 and 421 were identified as putative glycosyl hydrolases, by the presence of the comparable domains. DvM 421 is also a secreted peptide that is likely active in the lysosome. DvM 269 showed a match to a bacterial tetrahydofolate dehydrogenase as well as the presence of the THF-DHG domain. Finally, DvM 107 matched a galactosidase in *Strongylocentrotus purpuratus *as well as the relevant domain.

**Table 7:** Transcripts associated with carbohydrate digestion/hydrolases

**Transcript**	**Total**	**6 d fed**	**Unfed/2 d fed**	**Sig**	**Putative Function**	**Best match to NR protein database**	**E value**	GenBank*
	**Number of ESTs**							
DvM 63	2	2	0	Cyt	Glycosyl hydrolases	glucosidase II alpha subunit [S. purpuratus]	9.E-17	EU551634
DvM 107	2	1	1	Ind	galactosidase	galactosidase, beta 1 [S. purpuratus]	7.E-27	
DvM 269	1	1	0	Ind	C1-tetrahydrofolate synthase	methylene-tetrahydrofolate dehydrogenase	1.E-08	
DvM 421	1	1	0	Sig	hexosaminidase B	beta-N-acetylhexosaminidase [P. mammilata]	7.E-18	

*Accession number represents transcripts derived from this analysis and submitted to Genbank.

### Lipid binding

Table 8 lists the 11 transcripts for proteins probably associated with cell, protein and lipid binding functions. In contrast to the other protein classes, most transcripts in this category contained sequences from both the 6-day-fed and unfed/2-day-fed midguts. Included were four glycine rich proteins which appear to be most similar to salivary cement or glycine rich proteins, two BM86-like surface antigens, four mucin like proteins and a single transcript for a tick receptor outer surface protein A (TROSPA).

**Table 8:** Transcripts associated with Lipid

**Transcript**	**Total**	**6 d fed**	**Unfed/2 d fed**	**Sig**	**Putative Function**	**Best match to NR protein database**	**E value**	GenBank*
	**Number of ESTs**							
DvM 70	2	2	0	Cyt	BM86-like protein	Bm95 protein [B. microplus]	2.E-24	
DvM 558	1	0	1	Cyt	BM86-like protein	BM86-like protein [H. anatolicum]	1E-063	EU551651
DvM 613	1	0	1	SIG	glycine rich – cement like protein	hnRNP A2/hnRNP B1 [S. purpuratus]	5E-011	
DvM 527	1	0	1	Ind	glycine rich – cement like protein	glycine-rich protein [B. oleracea]	1E-026	
DvM 257	1	1	0	Ind	glycine rich – RIM36 like protein	cement protein RIM36 [R. append]	3E-046	EU551648
DvM 50	1	0	1	Cyt	cement protein RIM36/callogen	flagelliform silk protein [N. clavipes]	3E-024	
DvM 708	1	0	1	Cyt	TROSPA/cell wall protein	Hypothetical protein CBG01853	1E-011	
DvM 718	1	0	1	Cyt	mucin	mucin [H. longicornis]	5E-025	
DvM 635	1	0	1	Ind	mucin	CG4778-PA [T. castaneum]	4E-008	
DvM 827	1	0	1	SIG	mucin	CG4778-PA [T. castaneum]	7E-010	EU551635
DvM 20	6	4	2	Cyt	mucin	proline/threonine rich protein	2E-009	

*Accession number represents transcripts derived from this analysis and submitted to Genbank.

**Figure 17:**
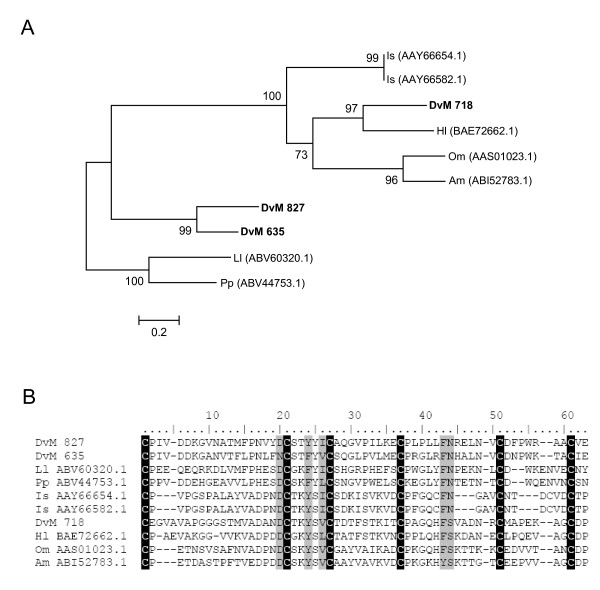
**Analysis of mucins.** (A) Phylogenetic tree based on maximum likelihood analysis of a *Dermacentor variabilis *midgut protein and published mucin-like protein sequences. The transcript identified in this analysis is in bold (DvM). Phylogenetic analysis was conducted on protein alignments using Tree Puzzle version 5.2. Values at nodes represent calculated internal branch node support (1000 replications). (B) Multiple sequence alignment (CLUSTALX) of protein sequences identified in a cDNA library of unfed/2 d fed or 6 d fed *D. variabilis *midguts (DvM) and published sequences found on Genbank. The conserved cysteines are highlighted. Shading represents 100% identity (black) or similarity (grey) among the sequences. Alignments were conducted using CLUSTALX. *D. variabilis *(Dv), *Ixodes scapularis *(Is), *Haemaphysalis longicornis *(Hl), *Ornithodoros moubata *(Om), *Argas monolakensis *(Am), *Lutzomyia longipalpis *(Ll), *Phlebotomus papatasi *(Pp).

The most abundant group associated with lipid binding contains four transcripts comprised of nine EST and is homologous to mucins. DvM 827 and 635 contain a putative secretory peptide while DvM 718 and 20 appear to be cytoplasmic proteins likely found on the cell membranes. Mucins are important in coating the lining epithelium of the gut, which contributes to their role as mucosal barriers. DvM 827 and 635 share identity with a conserved domain, CBM_14, a chitin binding peritrophin-A domain characterised by an extracellular domain that contains six conserved cysteines, forming three disulfide bridges (Figure 17b). DvM 20, derived from six ESTs, found in both the unfed/2-day-fed and 6-day-fed midguts, also shows a match to an allergen-like protein from the dust mite, *Dermatophagoides farinæ*. However, this sequence contains a conserved domain (Pfam01456) similar to mucin-like glycoproteins in the midgut peritrophic membrane of insects [52]. DvM 718, with one sequence found only in the unfed/2-day-fed midguts, shows a match to a mucin-like peritrophin sequence from the soft tick *O. moubata *as well as the chitin-binding Peritrophin-A domain, a characteristic feature of the peritrophic membrane (Figure 17a). The acellular peritrophic membrane, which forms within as little as 9 – 12 h after the commencement of feeding [57] lies close to the luminal surface of the epithelium where it protects the gut against injurious particulates and ingested microbes. It has been described from several species of ticks [58,59]. While DvM 718, 827 and 635 are closely related to other tick mucins, it is interesting to note how similar the mucins from *D. variabilis *midguts are with putative peritrophins from two sand fly species, *Lutzomyia longipalpis *and *Phlebotomus papatasi *(Figure 17a).

Glycine rich proteins were also abundant in this midgut category. Three of these putative proteins, DvM 50, 527 and 613, were found only in the unfed/2-day-fed midguts, while the fourth, DvM 257, was found only in the 6-day-fed midguts. DvM 613 was also a secreted protein. Each of the glycine rich proteins was similar to tick salivary gland cement-like proteins. Tick cement proteins are characterised by glycine rich aa repeats that occur as GLG [G/Y/S/F] tripeptides and may also show a GSPLSGF septapeptide [60]. All four of the putative glycine rich proteins showed variations of the tripeptides; DvM 257 also contained the septapeptide. DvM 257 showed 52% identity with RIM36, a cement protein from the salivary glands of *R. appendiculatus *(AAK98794.1) that contributes to the formation of the attachment cement. In contrast, DvM 527 and 613 showed the highest similarities, 39% and 40%, respectively, to a similar glycine rich cement protein from *I. scapularis*. The role of glycine rich proteins in the tick midgut is currently unknown.

Several transcripts were tentatively identified as membrane surface receptors/antigens. Two transcripts (DvM 70 and 558), show a match to Bm86, a membrane-bound surface protein from *Hyalomma anatolicum *(AAL36024.1) as well as Bm86 homologues from *R. microplus*, *H. longicornis *and *R. sanguineus*. Bm86 is similar to mammalian angiotensin-converting enzyme and, therefore, may function in a similar role in ticks [61]. Both appear to be glycoproteins; DvM 558 also shows a match for the von Willebrand factor, which contributes to platelet (thrombocyte) clumping. Both transcripts are 5' truncated by approximately 400 aa, yet along the approximately 232 aa of the 3' ends they are considerably conserved to other Bm86-like proteins found in ticks, particularly along the cysteine framework (data not shown).

A noteworthy finding was DvM 708, which showed a match for TROSPA, the *I. scapularis *midgut cell surface protein that binds to *Borrelia burgdorferi *spirochetes. This protein was found only in the unfed/2-day-fed ticks. Although the match is relatively poor, this finding may merit further study. To date, TROSPA has only been reported from *I. scapularis *[62].

### Transcripts related to Immunity within the midgut

**Figure 18:**
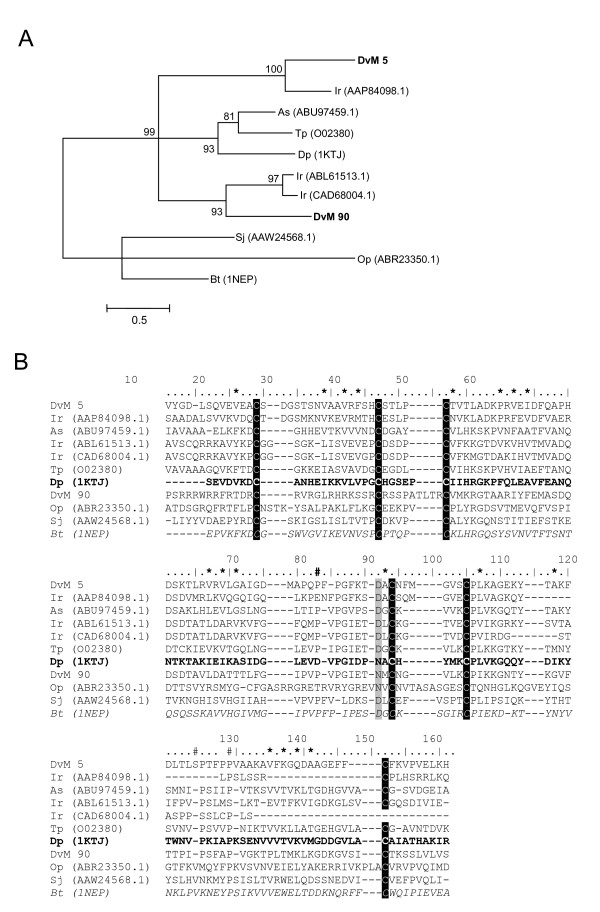
**Analysis of ML domain containing proteins.** (A) Phylogenetic tree based on maximum likelihood analysis of a *Dermacentor variabilis *midgut protein and published ML domain protein sequences. The transcripts identified in this analysis are in bold (DvM). Phylogenetic analysis was conducted on protein alignments using Tree Puzzle version 5.2. Values at nodes represent calculated internal branch node support (1000 replications). (B) Multiple sequence alignment (CLUSTALX) of protein sequences identified in a cDNA library of unfed/2 d fed or 6 d fed *D. variabilis *midguts (DvM) and published sequences found on Genbank. The conserved cysteines are highlighted. Number signs (#) represents putative cholesterol/lipid binding site sites based on the conserved domain of Niemann-Pick type C2 (Npc2) proteins (italisized). Asterisks (*) indicated amino acids involved in the putative lipid binding cavity based on ML domain of the dust mite allergen, Der P 2 (bold). Shading represents 100% identity (black) or similarity (grey) among the sequences. Alignments were conducted using CLUSTALX. *D. variabilis *(Dv), *Ixodes ricinus *(Ir), *Acarus siro *(As), *Schistosoma japonicum *(Sj), *Dermatophagoides pteronyssius *(Dp), *Ornithodoros parkeri *(Op), *Tyrophagus putrescentiae *(Tp), *Bos Taurus *(Bt).

Eight transcritps contained proteins putatively involved in immunity in the gut of the tick (Table 9). The most abundant transcripts in this group are putative allergen-like proteins containing ML domains (Figure 18). Two (DvM 5 and 90) of the five transcripts associated with allergen-like proteins were found in both the unfed/2-day-fed and 6-day-fed midguts, while DvM 339 and 378, both singletons, were expressed only in the 6-day-fed midguts; DvM 537, a singleton, was found in the unfed/2-day-fed midguts. DvM 90, present in both the unfed/2-day-fed and 6-day-fed midguts, is a secreted protein. The others are cytoplasmic or of indeterminate status. All five transcripts showed a match for the ML lipid recognition domain and all match allergen-like proteins or ML domains from other tick species, namely *I. ricinus *(AAP84098), which was found to be induced by a *Borrelia *infected bloodmeal in the midgut of this tick [63]. DvM 90, 339, 378, and 5 share significant homology with E1_DerP2_DerF2 protein domain (Pfam02221, E1_DerP2_DerF2) belonging to a family of ML domain-containing proteins that is a lipid recognition domain found in plants, fungi, animals and also includes the dust mite allergen Der P 2. These transcripts appear to code for a protein similar to the *Dermatophagoides pteronyssius *mite allergen (Pfam02221, DerP2_DerF2, ML domain), a lipid-binding protein which had the closest match to a similar protein in *Ixodes ricinus*, and other proteins of unknown function. These proteins are implicated in pathogen recognition, particularly recognition of pathogen-related lipids and involved in innate immunity and lipid metabolism [20]. Two transcripts, DvM 90 and 5, contain a ML lipid recognition domain also present in Niemann-Pick type C2 (Npc2)-type proteins and the phosphatidylinositol/phosphatidylglycerol transfer protein (PG/PI-TP). The latter is a ubiquitous cytosolic protein of eukaryotic cells that transports phospholipids from the endoplasmic reticulum and Golgi to other cell membranes [64]. Both transcripts were found in the clade with other ML domain containing acari species (Figure 18a). Allergen-like proteins are believed to be important in innate immunity by recognising and binding to lipids found on microbes, including pathogenic microbes ingested with the blood meal. Multiple pairwise alignment of tick ML domain-containing proteins revealed the conserved cysteine framework found in allergen-like proteins was also conserved in two *D. variabilis *derived sequences, DvM 5 and 90, as well as conserved aa involved in putative lipid binging activity (Figure 18b). In the context of the intracellular digestion of the bloodmeal, these ML-like proteins could be associated with lipid absorption form the phagolysosomes, a role that clearly needs more investigation.

**Table 9:** Contigs associated with Immunity

**Transcript**	**Total**	**6 d fed**	**Unfed/2 d fed**	**Sig**	**Putative Function**	**Best match to NR protein database**	**E value**	GenBank*
	**Number of ESTs**							
DvM 224	1	1	0	Cyt	dorin M precursor	OMFREP [O. moubata]	1E-005	
DvM 231	1	1	0	SIG	dorin M precursor	dorin M precursor [O. moubata]	1E-013	
DvM 97	2	1	1	Ind	macrophage MIF	Macrophage MIF [A. americanum]	3E-049	EU551650
DvM 5	28	21	7	Cyt	ML domain-containing protein	ML domain-containing protein [I. ricinus]	1E-028	EU551641
DvM 537	1	0	1	Cyt	ML domain-containing protein	ML domain-containing protein [I. ricinus]	0.35	EU551649
DvM 90	2	1	1	SIG	ML domain-containing protein	allergen like protein [I. ricinus]	2E-012	CAD68004
DvM 339	1	1	0	Ind	ML domain-containing protein	allergen like protein [I. ricinus]	2E-012	CAD68004
DvM 378	1	1	0	Ind	ML domain-containing protein	allergen Lep d 1.01	5E-007	

*Accession number represents transcripts derived from this analysis and submitted to Genbank.

The two transcripts tentatively identified as Dorin M-like proteins (DvM 224 and 231) show matches to a fibrinogen-domain containing lectin-like protein found in the hemolymph of *O. moubata *[65]; a similar lectin-like protein, Ixoderin, also occurs in *I. ricinus*. DvM 231 was found to show 25% identity to ixoderin and 22% and 24% identity, respectively, to the two lectins from *O. moubata*. These proteins are similar to fibrinogens and show the characteristic fibrinogen domain [66]. Notably, Ixoderin was also found in the midgut of *I. ricinus *[66]. DvM 231 also contained a signal peptide, similar to the other tick, insect and human lectins that were compared. This transcript was confirmed as a Dorin-M lectin by tryptic digestion/mass spectrometry (Figure 2). A second transcript (DvM 224) tentatively identified as a lectin, showed little similarity to any of the known lectins; its identity is uncertain. Lectins are believed to play an important role in antimicrobial activity in the hemolymph of ticks [66,67] as well as in the midgut of many blood-feeding insects [68].

**Figure 19:**
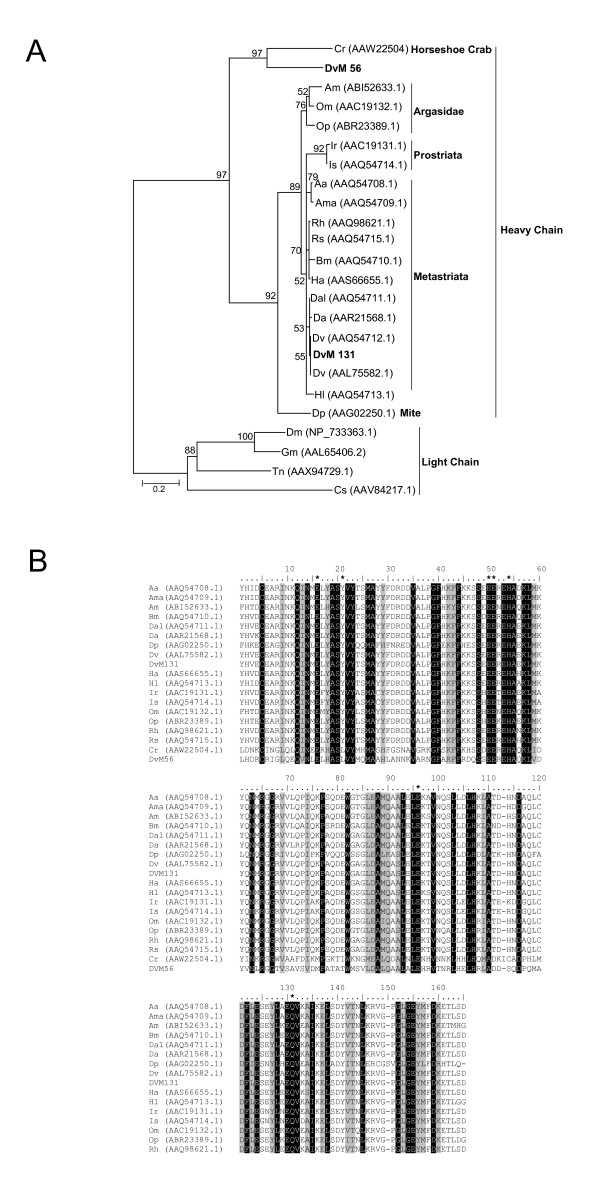
**Analysis of ferritin proteins.** (A) Phylogenetic tree based on maximum likelihood analysis of a *Dermacentor variabilis *midgut protein and published heavy and light chain ferritin protein sequences. The transcripts identified in this analysis are in bold (DvM). Phylogenetic analysis was conducted on protein alignments using Tree Puzzle version 5.2. Values at nodes represent calculated internal branch node support (1000 replications). (B) Multiple sequence alignment (CLUSTALX) of protein sequences identified in a cDNA library of unfed/2 d fed or 6 d fed *D. variabilis *midguts (DvM) and published heavy chain ferritins found on Genbank. Asterisks (*) represent the ferroxidase diiron center based on the ferritin conserved domain (cd00904). Shading represents 100% identity (black) or similarity (grey) among the sequences. Alignments were conducted using CLUSTALX.*D. variabilis *(Dv), *Argas monolakensis *(Am), *Ornithodoros moubata *(Om), *O. parkeri *(Op), *Ixodes ricinus *(Ir), *I. scapularis *(Is), *Amblyomma americanum *(Aa), *Amblyomma maculatum *(Ama), *Rhipicephalus (Boophilus) microplus *(Bm), *Hyalomma asiaticum *(Ha), *R. haemaphysaloides *(Rh), *Rhipicephalus sanguineus *(Rs), *Dermacentor variabilis *(Dv), *Dermacentor albipictus *(Dal), *Dermacentor andersoni *(Da), *Haemaphysalis longicornis *(Hl), *Dermatophagoides pteronyssinus *(Dp), *Carcinoscorpius rotundicauda *(Cr), *Drosophila melanogaster *(Dm), *Culicoides sonorensis *(Cs), *Trichoplusia ni *(Tn), *Glossina morsitans *(Gm).

The last protein we can include in this group is DvM 97, which is similar to the macrophage migration inhibitory factor (MIF) also found in the tick, *Amblyomma americanum *(E-value 3E-049), as well as many other invertebrates. This protein was found in both the unfed/2-day-fed and 6-day-fed ticks. MIF proteins have dopachrome isomerase activity, a function in arthropods provided by members of the Yellow family which is involved in melanization [69].

### Transcripts related to iron/heme metabolism and transport

Two transcripts tentatively identified as ferritins were recognised, both were found only in the unfed/2-day-fed midguts (Table 10) (Figure 19). DvM 56, with 3 ESTs, is a predicted secreted protein, while DvM 131, with 2 ESTs, is a predicted cytoplasmic protein. Both show an excellent match to similar sequences from *D. andersoni *and *D. variabilis*, as well as the conserved ferritin domain (cd00904). Ferritin is important as an iron storage reservoir and in protection against iron overload. Mosquitoes, for example, synthesise and secrete ferritin in response to iron exposure in direct relationship to iron dose [70]. A tick heavy chain ferritin was recently shown by Mulenga et al. [71] to be upregulated in response to mechanical injury and bacterial infection, thus suggesting its role in anti-microbial activity. Phylogenetic analysis of DvM 131 and 56 reveal the conserved nature of ferritins among tick species (Figure 19a and 19b). DvM 131 is found in the clade containing other *Dermacentor *species and is highly similar to a published ferritin from *D. variabilis *(AAL75582.1); therefore, this transcript does not appear to be a novel ferritin (Figure 19a). DvM 56 is found in the same clade as a horseshoe crab (*Carcinoscorpius rotundicauda*) ferritin which is ancestral to the tick-derived ferritins (Figure 19a). The placement of DvM 56 in this clade, yet separate from arthropod light chain ferritins, suggests that it is either a novel ferritin or is involved in a unique function, not previously identified. The conserved aa involved in the ferroxidase diiron center is conserved among the tick ferritins excluding DvM 56, in which the second aa of the domain is a lysine rather than a tyrosine, which may contribute to the divergence from other tick ferritins (Figure 19b). DvM 352 is tentatively assigned as an erythrocyte-binding protein, although the match is poor (E-value 0.027). Another transcript, DvM 342, tentatively assigned as a housekeeping protein, may actually function in erythrocyte binding since it shows a good match (E-value 1E-016) to the clathrin adaptor protein in *D. variabilis *and to the clathrin adapter domain in similar proteins found in insects and ticks. Clathrin-coated pits are essential for the binding and internalisation of hemoglobin by pinocytosis [72].

**Table 10:** Transcripts associated with iron/heme metabolism and transport

**Transcript**	**Total**	**6 d fed**	**Unfed/2 d fed**	**Sig**	**Putative Function**	**Best match to NR protein database**	**E value**	GenBank*
	**Number of ESTs**							
DvM 352	1	1	0	Ind	erythrocyte binding protein	hypothetical protein [R. norvegicus]	0.001	
DvM 56	3	0	3	SIG	Ferritin	ferritin heavy chain-2 [C. rotundicauda]	2E-043	
DvM 131	2	0	2	Cyt	Ferritin	ferritin [D. variabilis]	3E-094	

*Accession number represents transcripts derived from this analysis and submitted to Genbank.

### Secreted proteins similar to tick salivary proteins

Five transcripts were similar to proteins secreted in the saliva of ticks. Each transcript contained a significant match to a protein identified from a tissue-specific cDNA library of dissected salivary glands. DvM 682, 830, 551, and 134 were found only in the unfed/2-day-fed midguts, whereas DvM 64 was found in the 6-day-fed guts. Two transcripts (DvM 682 and 134) had clear signal peptides, whereas the putative secretion of DvM 830, 551 and 64 is based on homology to other known secreted proteins.

### Unknown, unassigned transcripts

This group comprised transcripts that had low confidence matches in the NR and/or ACARI databases, or both, or had conflicting tentative assignments with relatively low matches. Although many were short and incomplete sequences, others were long or appeared to be complete sequences and may represent novel genes. Many (DvM 37) were secreted proteins. The reader may access these sequences for further study accessing the the supplemental data given in the Methods section of this report.

### Protein identifications

Tryptic digestion/mass spectrometry (mass fingerprinting) (Figure 2) provided supporting evidence for the functional assignment of the following transcripts found in the midgut library: 1) 70 kD heat shock protein; 2) GST (*D. variabilis*); 3) Cytochrome b5 (*I. scapularis*); 4) histone *(I. scapularis*); 5) paramyosin (*Sacrcoptes scabei, R. microplus*); and 6) BM86 membrane glycoprotein (*R. microplus; H. anatolicum anatolicum*); and 7) glycogen phosphorylase (DvM 100, *Syntrophus aciditrophicus *gi|85858688). Other proteins found in the tryptic digest but not in the cDNA library were: 1) prenyl cysteine oxidase; 2) a statin-related protein (*Rattus norvegicus*); and 3) a hemelipoglycoprotein precursor (*D. variabilis*) (determined by RTB, NIAID, NIH sequencing facility). The latter is noteworthy in view of reports of the role of the female tick midgut as a secondary site of synthesis of vitellogenin, a known hemelipoglycoprotein, as well as in the fat body [73]. Attempts to identify proteins isolated from PVDF blots by Edman degradation were unsuccessful because they were N-terminally blocked.

## Conclusion

In this study, we constructed a PCR-based cDNA library from the midguts of unfed/2-day-fed and 6-day-fed virgin females of the tick *D. variabilis *and analysed 1,679 high-quality randomly sequenced ESTs. We obtained partial or full-length information on proteins and peptides, most of which appear to be novel. No transcripts were found from rabbits, the host on which the ticks had been fed. In addition to functional assignments based on the criteria described previously, we confirmed the identity of seven proteins by tryptic digestion/mass fingerprinting of bands eluted from SDS-PAGE gels of midgut extracts, as well as four other proteins for which no transcripts were found.

Comparison of the transcripts from the unfed/2-day-fed versus the 6-day-fed ticks indicates that most of the sequences were found in the latter; few were found in both. This was especially true for the peptidases; 17 of the 26 transcripts in this category were expressed in the 6-day-fed midguts, and only three appeared in both. The same pattern is evident for the peptidase inhibitors (6 versus 3), hydrolases (6 versus 1) and oxidative stress proteins (12 versus 4, with only 1 found in both). This is not surprising, since little blood is imbibed during the first two days after attachment. However, several functionally important proteins and peptides were found to have been expressed in the early feeding period, especially 1) the cell/protein/lipid-binding proteins that include erythrocyte and hemoglobin-binding proteins; eight of these were found in the unfed/2-day-fed midguts and one were found in both; 2) peritrophin, responsible for formation of the peritrophic membrane, but absent in the later feeding period; and 3) cysteine and metallo-peptidases, peptidase inhibitors and oxidative stress proteins, many of which may have an antimicrobial role.

The library shows a remarkable degree of redundancy, a finding previously reported for the salivary gland cDNA library [74-76]. Especially noteworthy is the large number of cysteine and serine peptidases, serpins, hydrolases, GSTs, dehydrogenase and other free-radical reducing enzymes, and the numerous binding proteins. Such duplication is consistent with the finding of extensive gene duplication for genes of great functional value in the blood feeding process, as suggested by Ribeiro et al. [77]. Only 58 of the 835 (6.9%) transcripts showed signal peptides, indicating that most were involved in intracellular processes in contrast to the salivary glands, where a much higher proportion (29%) comprised secreted proteins [77,78]. Less than half had functional assignments based on the criteria described above. The large number of peptidases and peptidase inhibitors is consistent with the intracellular digestive processes characteristic of these ticks.

As might be expected, the largest category of transcripts in the library, with 289 transcripts, comprised the housekeeping proteins, subdivided into a least 23 functional groups. Most represented transcripts for RNA structural proteins, transcription/translational activity, metabolism, cellular and mitochondrial oxidative respiration, antioxidant activity, proteasome machinery, and other housekeeping activities. The remaining non-housekeeping proteins were much less numerous. Of special interest are the proteins concerned with blood-meal digestion, especially the peptidases and peptidase inhibitors. When ticks feed and ingest blood, the hemoglobin liberated from lysis of the erythrocytes binds to the luminal surfaces of the midgut epithelial cells. There it is absorbed and incorporated into phagolysosomes where it is digested [72]. Studies by more recent workers have shown the presence in the tick midgut of cysteine peptidases [69], unidentified aspartic and cysteine peptidases [79] and uncharacterised cysteine and serine peptidases [80]. Aside from the serine peptidases from the midguts of *R. appendiculatus *and *H. longicornis *[16,17,81] and an aspartic peptidase from *H. longicornis *[54], none were sequenced or their molecular structure identified. These enzymes showed greatest activity at acid pHs, suggesting that they are lysosomal peptidases. However, none have reported as many different peptidases as were recognised in the *D. variabilis *cDNA midgut library described in this paper. Only four, transcrips DvM 13, 254, 314 and 594 were secreted proteins, indicating that most of these peptidases were functioning in intracellular protein digestion, presumably digestion of endocytosed hemoglobin within the digestive cells. Three of the cysteine peptidases, DvM 62, 96, and 694, included a conserved domain for the legumain-like peptidase associated with hemoglobinase, further supporting the putative role of these peptidases in blood-meal digestion. Others, such as aspartic peptidases and serine peptidases also were found without signal peptides, suggesting they are concerned within intracellular protein digestion (primarily hemoglobin) in the phagolysosome.

Metallopeptidases are also of interest, of which four were found in the midgut library. Evidence from insects suggests that they may be important in cellular immune defense [82]. Interestingly, a metallopeptidase from the tick *I. scapularis *was shown to contain fibrin and fibrinogen activities, revealing yet another potential function of the metallopeptidases found here [83]. Similarities with known peptidases in other ticks, other acarines and insects were relatively low (31% or less), suggesting that most of these midgut peptidases are novel. Another noteworthy finding is the large number of transcripts known to function as antimicrobial agents, e.g., pathogen-recognition proteins, or in a dual role, e.g., as oxidative stress reduction and innate immune peptides. In haematophagous insects, microbes ingested with the blood meal provoke an effective defense by upregulating lectins, lysozyme, defensins, cercropins and other antimicrobial peptides [78,84,85]. This study suggests that an exceptionally large number of innate immune peptides or proteins occur in the midgut. Among these are the nine different peptidase inhibitors found in the *D. variabilis *midgut library. Although many peptidase inhibitors have been reported from ticks, most were from the salivary glands or hemolymph. A search of the literature showed four serpins in *R. appendiculatus *expressed in various tick organs in addition to salivary glands and midguts [86] but only one from the midgut of *H. longicornis *[74]. A cystatin reported from the midguts and hemocytes of *H. longicornis *[75] was found to increase up to 1.8 times greater when the ticks were exposed to *Babesia gibsoni *or *B. bovis*, while the recombinant protein inhibited growth of *B. bovis *grown in culture. The finding of numerous transcripts for serpins and cystatins in the *D. variabilis *midgut suggests an important role for those proteins in innate immune defense. Other proteins expressed during blood feeding probably play a role in defense against microbial invasions. Included is the von Willebrand factor peptide, an anti-clotting factor that also occurs in *I. ricinus *after *B. burgdorferi *challenge [63], the ML-domain proteins and the lectins. ML-domain proteins function as pathogen recognition proteins. The 6-day-fed midguts were also found to contain two sequences for Dorin M lectin-like peptides. Several lectins have also been reported from ticks, including Dorin M from the hemolymph of *O. moubata *[65] and Ixoderin from the salivary glands and midgut of *I. ricinus *[67]. Lectins bind to sialic acid, hexosamines and other compounds characteristic of the cell walls of bacteria and fungi and, consequently, they are important in defence against invading microbes and preventing pathogen/parasite transmission.

It has now become increasingly clear that the midgut of ticks presents a hostile environment for ingested microbes. This is evident not only because of the presence of the more familiar antimicrobial peptides (lysozyme and defensin), but also because of the expression of oxidative stress-reducing, detoxifying and lipid- or protein-binding peptides that confer antimicrobial properties, e.g., GSTs, metallothioneins, peroxiredoxins, midgut lectins and a large number of peptidases. Microbial infection is known to cause oxidative stress, leading to upregulation of GSTs, peroxiredoxins and other oxidative stress-reducing proteins [76]. Clearly, the arsenal of antimicrobial agents is considerably more extensive than just defensin or lysozyme, which are either poorly expressed or absent. Indeed, in *D. variabilis*, the midgut lysozyme message was not significantly upregulated by blood-feeding [87] and no evidence of either the lysozyme or defensin peptides was observed in other studies of the midgut of this tick [3], although it is strongly upregulated following the blood meal in *O. moubata *[88]. However, it is possible that defensin peptide was overlooked. This peptide is known to bind with a serine peptidase to form an SDS-stable complex with an apparent molecular weight > 26 kDa in the blood-sucking fly *Stomoxys calcitrans *[89]. In addition to the native tick proteins, fragments of hemoglobin digestion also contribute to destruction of ingested microbes [1-3]. Bacteria such as *Bacillus subtilis*, *Escherischia coli *and *Borrelia burgdorferi*, fed to *D. variabilis *females by capillary oral feeding were taken into the midgut, but none could be re-cultured within 24 h after exposure. When midguts of these ticks were examined by electron microscopy, intact *B. burgdorferi *and rod-shaped bodies resembling *E. coli *were found in several samples [81]. Since lytic activity does not appear to be the primary antimicrobial response, it is interesting to speculate that the Dorin M-like lectin and the several secreted cysteine and aspartic peptidases noted previously may kill or at least inhibit the survival of these organisms in the midgut lumen. In contrast to *D. variabilis, E. coli *cells ingested into the midgut of the soft tick, *O. moubata*, were found to survive in the midgut lumen for up to 20 days, and were destroyed gradually only after they were endocytosed within the midgut epithelial cells [90]. Within the midgut lining cells, the upregulation of lectins or lectin-like proteins, numerous GSTs, aldehyde dehydrogenase, metallothionein, SOD and other oxidative stress enzymes in response to hemoglobin uptake and digestion would also contribute to the destruction of invasive microbes.

The authors recognise that the cDNA midgut library is an exploratory catalogue of transcripts expressed in this tissue in feeding females and is most likely incomplete. Although functional assignments could be made for approximately 45% of the concensus sequences and singletons, the remainder could not be assigned, often because they were not full-length sequences. Other classes of proteins and peptide transcripts previously reported in the midgut were not recognised, e.g., defensin [3,83], lysozyme [87], heme transferases and integrins. This annotated catalogue of midgut transcripts from the midgut of a blood-fed hard tick may be useful to scientists wishing to investigate the role of tick midgut in bloodmeal digestion as well as its ability to cope with oxidative stress, antimicrobial activity or the passage of the tick-borne pathogens acquired during blood feeding. Most of the 418 putatively identified transcripts expressed in this library appear to be novel. Similarly, the remaining unidentified transcripts also lack significant similarities to known tick or insect proteins. This report makes all available for inspection and further study.

## Methods

### Solvents and organic compounds

Water was 18 MΩ quality produced on site using a MilliQ water purification system (Millipore, Bedford, MA, USA). Organic compounds were obtained from Sigma Chemical Corporation (St. Louis, MO, USA) or as stated.

### Ticks

*Dermacentor variabilis *was colonised and maintained as described previously [84]. Two groups of virgin female ticks were used to create the cDNA midgut library. Briefly, group 1 comprised blood-fed ticks that had fed 6-day on New Zealand white rabbits (*Oryctolagus cunniculus)*; group 2 comprised ticks that had fed 2-day on rabbits, along with unfed females. All use of animals for this research was done in accordance with protocols approved by the Old Dominion University Institutional Animal Care and Use Committee (IACUC). The approved protocols are on file in the Old Dominion University Animal Care Facility Office.

### Tissue collection and cDNA library construction

Blood-fed virgin female ticks were detached from their rabbit hosts, surface-sterilised with 3% H_2_O_2 _and 70% ethanol and dissected to expose the midguts. For group 1, samples of midgut tissues were excised from five females, washed 1 × with phosphate-buffered saline (PBS, pH 7.2) and immersed in RNAlater (Ambion, Austin, TX) at 4°C until needed. For group 2, sample midgut tissues were excised from ten 2-day-fed females and ten unfed females, washed as described above, combined and immersed in RNAlater. The Micro-Fast Track mRNA isolation kit (Invitrogen, San Diego, CA, USA) was used to isolate mRNA in accordance with the manufacturer's instructions with some modification [78]. Briefly, the SMART cDNA library construction kit (Clontech, Palo Alto, CA, USA) was used to create the PCR-based cDNA library. A 100 ng of mRNA was reverse transcribed using the SMART PowerScript™ reverse transcriptase and CDS III/3' PCR primer (Clontech) for 1 h at 42°C. Second strand synthesis was done with a PCR-based protocol using the SMART 5' PCR primer (Clontech) as the sense primer and CDSIII/3' PCR primer as the antisense primer. These primers create S*fi*I A and B sites as the ends of the nascent cDNA. Double-stranded (ds) cDNA synthesis was carried out using a Perkin-Elmer 9700 Thermal cycler (Perkin Elmer Corp., Foster City, CA, USA) using the Advantage 2 *taq-*Polymerase (Clontech). PCR conditions were as follows: 95°C for 1 min, then 8 cycles at 95°C for 10 s and 68°C for 6 min, allowed to cool (4°C) and a 3 μl aliquot removed and stored. PCR amplification was repeated and additional aliquots removed after every 2 cycles until a total of 18 cycles had elapsed. The aliquots from each group of cycles were run on an agarose gel with Ethidium Bromide (EtBr) and the optimum number of cycles that would avoid over amplification of the most abundant cDNAs was determined. The double-stranded cDNA was treated with peptidase K (0.8 μg/μl) and washed (H_2_O) 3 × using 100 mol wt cut off (MWCO) Amicon filters (Millipore). After cleaning, the double-stranded cDNA was digested with S*fi*I enzyme at 50°C and the cDNA fragments were fractionated using the Chroma spin™ columns provided (Clontech). Fractions were separated into three different sizes (large, medium and small), concentrated, washed (H_2_O), filtered (100 kDa Amicon filters), concentrated and ligated into a λ-TriplEx-2 vector (Clontech). The ligation reaction product was packed using the Gigapack Gold III kit (Stratagene, Cedar Creek, TN, USA) according to the manufacturer's instructions. The resultant library was plated by infecting log-phase XL-1 blue cells (Clontech). The amount of recombinants was determined by PCR using the vector primers flanking the inserted cDNA and then visualised on agarose gels (ethidium bromide).

### Sequencing the D. variabilis midgut library

The cDNA libraries from the two groups were plated (200 plaques/150 mm plate). Plaques were picked at random and transferred to the wells of a 96-well propylene plate containing 75 μl/well. Bacteriophage (4 μl), forward and reverse primers, sequencing reactions and cleanup were done as described previously [78]. Cleaned PCR products were used as a template for cycle-sequencing reactions using BigDye Terminator v3.1 cycle sequencing kit (Applied Biosystems, Foster City, CA). Sequencing reactions were cleaned using a multiscreen 96-well plate cleaning system (Millipore, Billerica, MA). Dried samples were resuspended with 25 μl of deionised, ultrapure, formamide. Samples were directly sequenced on an ABI 96 capillary DNA sequencer (Applied Biosystems, Foster City, CA) or stored at -80°C.

### Bioinformatics analysis

A detailed description of the bioinformatic treatment of the data appears in Valenzuela et al. [85]. Data manipulation was preformed using a set of customised executable programs written in Visual Basics by José M.C. Ribeiro at the National Institutes of Health. Primer and vector sequences were removed from the raw sequences and the resultant (cleaned) sequences were compared against the NR protein database using an executables program obtained at the NCBI FTP site [91]. Related sequences were grouped into clusters and aligned using CAP3 sequence assembly program [92]. Using the appropriate BLAST algorithm (BLASTX, BLASTN or RPS-BLAST), individual concensus sequences and singletons were compared to the NR protein database of the NCBI, the GO fasta subset [93], CDD of NCBI [20] containing the KOG [94], Pfam [95] and SMART [96] protein motif databases [18] and to custom-downloaded databases containing the ACARI (a subset containing mite and tick sequences), mitochondrial/plastid and rRNA nucleotide sequences available at the NCBI. We submitted all translated sequences (starting with a Met) to the Signal P server [97] to detect signal peptides indicative of secretion. The three-frame translation of each dataset was used to determine open reading frames (ORF). Only ORF that started with a methionine and were longer than 40 amino acid (AA) residues were submitted to the SignalP server. The grouped and assembled sequences, BLAST results and signal peptide results were combined in an Excel spreadsheet and manually verified and annotated. A stand alone file which the user should download and extract is found at: . Additionally, a hyperlinked excel file can be obtained from: 

### Phylogenetic analysis

For phylogenetic analysis of transcripts of interest, the consensus sequence was translated into the appropriate open-reading frame using EditSeq software (DNAStar). Related protein sequences were downloaded from NCBI and aligned using ClustalX [98]. The resulting alignment was manually refined and trimmed using BioEdit sequence-editing software [99]. Alignments were then submitted to ProtTest version 1.2.6 [100] to determine the best-fit model protein substitution for each particular alignment. Phylogenetic analysis was conducted on protein alignments using Tree Puzzle version 5.2 [101] incorporating the predicted model of evolution as defined by ProtTest. Tree Puzzle constructs phylogenetic trees by maximum likelihood using quartet puzzling, automatically estimating internal branch node support (1000 bootstrap replications) [102]. Trees were visualised and annotated using Mega 4.0 [103]. The names of the sequences in the alignments and phylograms were abbreviated with the species initials and GenBank accession number; for the transcripts from the cDNA libraries, the transcript number was added.

### Supplemental material

To save space and simplify reading, many of the supporting figures are located in the form of supplemental materials hyperlinked throughout the paper either to the NCBI page [104] where this and other transcriptomes are located or to the publisher's archives associated with this paper. Readers may access these materials by clicking on the hyperlinked files.

### Protein identification

Blood-fed female ticks were detached, surface washed with 70% ethanol: 3% H_2_O_2 _to remove contaminants. The midguts were removed, washed in PBS buffer and homogenised in cold (4°C) in lysis buffer containing 20 mM Tris-HCL, 137 mM NaCl, 2 mM EDTA, 0.1% Triton-X 100 and 10% glycerol [105], supplemented with 0.1 – 0.2 mM PMSF (Sigma, St. Louis, MO, USA) and a 200-fold dilution of peptidase inhibitor cocktail (Sigma). The extract (N = 25) was sonicated, centrifuged at 14,000 × *g*, and then frozen (-20°C) until needed. Bradford protein assays were performed as described by the manufacturer (BioRAD, Richmond, CA) using immunoglobulin G as the standard. Samples (~40 μg per lane) of the midgut protein extract were electrophoresed using NuPage 4 – 12% 1 mm thick gradient gels under reducing conditions in accordance with the manufacturer's instructions (Invitrogen). Relative mol wts were estimated using pre-stained SeeBlue™ and unstained Mark 12™ mol wt markers (Invitrogen). For amino-terminal sequencing of the midgut proteins, the proteins were electro-blotted to PVDF blot paper in a Xcell II Mini-Cell with transfer buffer (Invitrogen) and the membrane stained with 0.025% Coomassie Blue (R) in the absence of acetic acid. Stained bands were cut from the blot membrane and subjected to Edman degradation in a Procise sequencer (Perkin Elmer Corp.). The resultant AA sequences were searched against the most likely protein translations of each cDNA sequence obtained in the mass sequencing project, as described previously [78]. Identifications were also done by tryptic digestion/mass fingerprinting of selected gel slices excised from the protein gels at the University of Virginia's Keck Biomolecular Research Facility as described previously [3,80], and at the Proteomics Facility, Research Technology Branch, National Institute of Allergy and Infectious Diseases. The sensitivity of detection would detect proteins or peptides even when present in concentrations as low as 1% of the sample.

## Abbreviations

aa, AA: amino acid; ACARI: tick, mite classification; CDD: conserved domains database; EST: expressed sequence tags; GO: gene ontology; GRX: glutaredoxin; GST: glutathione-S-transferase; KOG: EuKaryotic orthologous groups; MIT-PLA: mitochondrial-plastid; ML: metabolic lipid; NCBI: National Center for Biotechnology Information; NR: non-redundant; PCR: polymerase chain reaction; Pfam: protein families and domains; SMAIL/TRAIL: amino acid conserved string; SMART: simple modular architecture research tool; SOD: superoxide dismutase; TRX: thioredoxin.

## Authors' contributions

JMA participated in all aspects of this manuscript including the conception and coordination of the study, construction and sequencing of the cDNA libraries, bioinformatics analysis and manual annotation of sequences, alignments and phylogenetic analysis of transcripts and drafting the manuscript. DES contributed to the conception and coordination of the study, rearing and dissection of the ticks used in the study, construction of the cDNA libraries and drafting the manuscript. JGV participated in the conception and coordination of the study and drafting the manuscript. All authors have read and approved the final manuscript.
